# Oligomerization enables the selective targeting of an intrinsically disordered region by a small molecule

**DOI:** 10.1126/sciadv.adz7400

**Published:** 2026-02-27

**Authors:** Stasė Bielskutė-García, Borja Mateos, Muhammad Awawdy, Carla Garcia-Cabau, Henri Niskanen, Carolina Sánchez-Zarzalejo, Lorenzo Bracaglia, Roberta Pierattelli, Isabella C. Felli, Marta Frigolé-Vivas, Jesús García, Antoni Riera, Denes Hnisz, Xavier Salvatella

**Affiliations:** ^1^ Institute for Research in Biomedicine (IRB Barcelona), The Barcelona Institute of Science and Technology, Baldiri Reixac 10, 08028 Barcelona, Spain.; ^2^ Max Planck Institute for Molecular Genetics, Ihnestraße 63-73, 14195 Berlin, Germany.; ^3^ CERM and Department of Chemistry “Ugo Schiff,” University of Florence, Via Luigi Sacconi 6, 50019 Sesto Fiorentino, Florence, Italy.; ^4^ Inorganic and Organic Chemistry Department, University of Barcelona, Martí i Franquès 1-11, 08028 Barcelona, Spain.; ^5^ ICREA, Passeig Lluís Companys 23, 08010 Barcelona, Spain.

## Abstract

Intrinsically disordered regions (IDRs) in proteins are increasingly recognized as attractive targets for therapeutic intervention. A number of small molecules interacting with IDRs have been identified, but the lack of persistent secondary and tertiary structure of these regions has led to the prevailing view that they cannot be targeted selectively. Here, we show that a small molecule targeting an IDR evaluated in a clinical trial interacts selectively with an oligomeric form of its target, which is more structured than the monomer and is stabilized by interactions involving aromatic residues in partially α-helical regions. The interaction reshapes the conformational ensemble of the target, alters the biophysical properties of its phase-separated condensates in vitro, and attenuates RNA polymerase II recruitment in cells. Our findings provide mechanistic insights into how small molecules can selectively recognize IDRs.

## Teaser

The druggability of intrinsically disordered regions.

## INTRODUCTION

The intrinsically disordered regions (IDRs) of proteins represent highly attractive targets for therapeutic intervention (*1*). However, they are often considered undruggable because they do not form stable secondary and tertiary structures, which are required for small molecules to interact with them selectively (*2*, *3*). Several compounds targeting IDRs have demonstrated selective inhibition in preclinical studies, but the molecular basis for selectivity remains poorly understood (*4*–*10*). To address this question, we investigated the interaction between a small molecule targeting an IDR that reached clinical trials, EPI-001, and its target, the activation domain (AD) of the androgen receptor (AR). Here, AR AD defines the intrinsically disordered N-terminal domain of AR (residues 1 to 558), a therapeutic target for castration-resistant prostate cancer, a late stage of prostate cancer that remains incurable (*11*, *12*).

We found that EPI-001, initially identified in a phenotypic screen and characterized as a selective AR AD inhibitor in preclinical studies (*12*), interacts selectively with a transient oligomeric form of the AR AD, which is more structured than the monomer and is stabilized by interactions between aromatic residues in regions of sequence partially folded as α helices. Our findings also revealed that the interaction with EPI-001 modifies the conformational ensemble of the AR AD and the physical properties of the phase-separated state in vitro as well as reduces the ability of AR to recruit RNA polymerase II (RNAPII) in cells. In summary, our results help explain how small molecules can target IDRs with selectivity by exploiting their propensity to transiently form partially folded oligomers.

## RESULTS

### EPI-001 selectively interacts with oligomeric AR AD

**Fig. 1.**
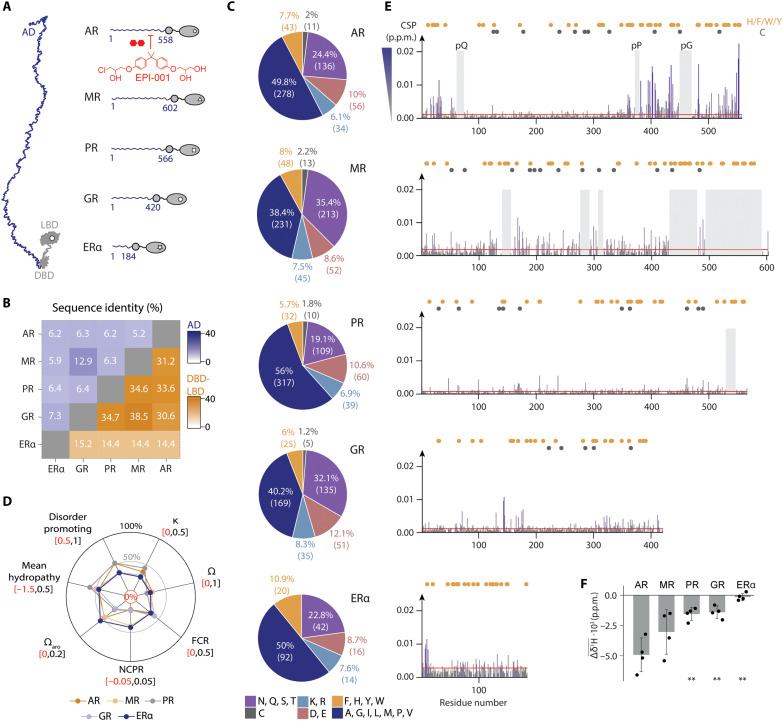
EPI-001 interacts selectively with the AD of AR. (**A**) The selectivity panel is composed of NRs activated by steroid hormones, having a long variable AD followed by conserved globular domains (DBD and LBD). (**B**) Sequence identity fraction of the ADs and DBD-LBD in blue and orange, respectively. (**C**) Amino acid composition fractions for six different residue types (polar, purple; Cys, gray; positive charge, light blue; negative charge, pink; aromatic, orange; hydrophobic, dark blue). (**D**) Overall sequence properties for the different ADs. κ and Ω values describe the segregation of charged and proline residues, respectively. Ω_aro_ represents the aromatic clustering. FCR, fraction of charged residues; NCPR, net charge per residue. (**E**) CSPs extracted from two-dimensional (2D) ^1^H-^15^N NMR correlation spectra of the corresponding AD induced in the presence of 10 molar equivalents of EPI-001, where p.p.m. stands for part per million. Orange and gray circles indicate the position of aromatic and Cys residues, respectively. The red line represents the significant threshold calculated as the mean plus five SDs of the first quartile of CSPs. Gray shaded boxes indicate residues unassignable in the apo spectra due to intrinsic broadening or overlap and therefore not evaluated for CSPs; these regions remain unassigned upon EPI-001 addition. (**F**) Average ^1^H shifts from distinctive EPI-001 signals in the presence of 0.1 molar equivalents of the corresponding AD. The error bar denotes the SD (*n* = 4).

To investigate the molecular basis of the selective inhibition of AR transcriptional activity by EPI-001, we examined the interaction of this small molecule with the ADs of four nuclear receptors (NRs) other than AR: the mineralocorticoid (MR), progesterone (PR), glucocorticoid (GR), and estrogen (ERα) receptors (Fig. 1A) (*13*, *14*). Here, for consistency with our previous work (*15*), we use the term AD to refer to the intrinsically disordered N-terminal domain of each receptor rather than to sequence motifs involved in specific interactions. NRs are transcription factors with conserved domain structures that are drug targets across various disease areas (*16*, *17*). We chose these NR ADs as a selectivity panel due to their similar biological activities—the recruitment of the transcription machinery via the formation of transcriptional condensates (*15*, *18*)—and sequence properties. While sequence alignment alone does not immediately reveal sequence similarities (Fig. 1B), they become clear in parameters such as amino acid composition (Fig. 1C) and sequence patterning (Fig. 1D and fig. S1, A and B), which differ from those of IDRs not belonging to the panel such as hnRNPA1 (heterogeneous nuclear ribonucleoprotein A1) and FUS (fused in sarcoma) (fig. S1C) (*19*–*21*).

Solution nuclear magnetic resonance (NMR) spectroscopy revealed that the spectra of the NR ADs, like that of the AR AD, have low chemical shift dispersion and a wide range of signal intensities, as expected in a disordered protein displaying transient long-range interactions (fig. S1, D and E) (*22*–*25*). We found that the chemical shift perturbations (CSPs) caused by EPI-001 in the NR AD spectra were much weaker than those observed for the AR AD (Fig. 1E and fig. S1F) (*9*, *15*). In the MR AD, in the absence of EPI-001, many resonances were broadened beyond detection, and those that remained detectable were not altered by addition of this small molecule. To rule out that EPI-001 interacts with residues undetected in the spectra, we added MR AD to a solution of the AR AD interacting with this small molecule and observed no signs of competition (fig. S1, G and H). Although CSPs are not absolute measures of the strength of intermolecular interaction, our findings suggest that EPI-001 is a selective ligand of the AR AD (Fig. 1F and fig. S1I).

**Fig. 2.**
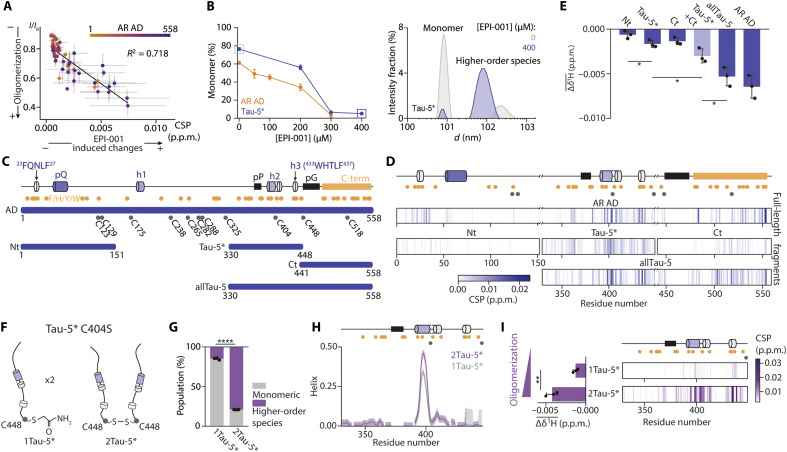
EPI-001 interacts with oligomeric AR AD. (**A**) Correlation between signal decrease upon oligomerization (*15*) and CSPs induced by EPI-001 in the AR AD. (**B**) (Left) Percentage of monomer in 35 μM AR AD and 400 μM transactivation unit 5 (Tau-5*), a subregion of the AR AD, across EPI-001 titrations. The remaining percentage represents high-order species by DLS. (Right) DLS intensity measurement of Tau-5* in the absence (gray) and presence (blue) of 400 μM EPI-001. (**C**) Schematic of AR AD fragments. Helical motifs are represented as cylinders (fig. S2F) and proline and glycine homorepeats as black squares. Orange and gray circles indicate aromatic and Cys residues, respectively. (**D**) CSPs barcode plots from 2D ^1^H-^15^N NMR correlation spectra of 25 μM AR AD and its fragments in the absence and presence of 250 μM EPI-001. (**E**) Average of the ^1^H shifts induced on three distinct EPI-001 signals in the presence of 0.1 molar equivalent of AR AD and its fragments. The Tau-5* + Ct lighter blue bar represents the sum of the independent Tau-5* and Ct experiments. (**F**) 2Tau-5* is formed by a disulfide bond at C448, while in 1Tau-5*, C448 is blocked with iodoacetamide to prevent disulfide bond formation. (**G**) Percentage of monomeric and high-order species in 1Tau-5* and 2Tau-5* by DLS at a concentration of 2.44 mg/ml. Error bars indicate SDs (*n* = 3). (**H**) NMR-derived helical content of 1Tau-5* and 2Tau-5* at 0.31 mg/ml, using the δ2D algorithm (*71*). (**I**) (Left) Average shift across three EPI-001 ^1^H signals at 250 μM induced by 1Tau-5* or 2Tau-5* (0.31 mg/ml). (Right) Per-residue CSPs in 2D ^1^H-^15^N NMR correlation spectra of 1Tau-5* or 2Tau-5* (0.31 mg/ml) after adding 250 μM EPI-001. In both cases, the ratio corresponds to 1:10 of EPI-001 interaction sites on the protein to EPI-001.

EPI-001 perturbs resonances of the AR AD (*9*, *15*) that decrease in intensity upon AR AD oligomerization (*15*, *26*), suggesting that the CSPs caused by this small molecule could be, at least partially, indirect and the result of an increased oligomerization (Fig. 2A). We thus used dynamic light scattering (DLS) to analyze the oligomerization propensity of the NR ADs, obtaining that the AR, ERα, GR, and PR ADs had similar oligomerization propensities, all lower than that of the MR AD (fig. S2, A to C), a result that can explain the line broadening observed in the NMR spectra of the MR AD (Fig. 1E and fig. S1D) (*15*). In addition, we found that the population of AR AD monomers decreases monotonically with the concentration of EPI-001 (Fig. 2B) whereas that of the MR AD, the member of the selectivity panel with the highest oligomerization propensity, does not. The oligomerization equilibrium of the other members of the selectivity panel was affected by EPI-001 but to a lesser extent than that of the AR AD (fig. S2D). These results indicate that this small molecule selectively stabilizes the oligomers formed by the AR AD.

We next carried out experiments to investigate whether EPI-001 is selective for a specific subregion of the AR AD. The CSPs appear to be a convolution of direct chemical shifts, caused by contacts between target and ligand, and indirect ones, caused by changes in the structure or degree of oligomerization. To increase our chances of detecting direct CSPs and thus identify the site of interaction, we studied the CSPs induced by EPI-001 in AR AD fragments including Nt (residues 1 to 151) (*27*), transactivation unit 5 (Tau-5*; residues 330 to 448) (*9*)—a subregion of the AR AD that plays a key role in prostate cancer cell growth under therapy-resistant conditions (*28*)—and Ct (residues 441 to 558) (Fig. 2C and fig. S2, E and F) (*15*), which collectively encompass the regions where CSPs are observed in the full-length AD (Fig. 1E). We also studied the CSPs induced in an additional fragment allTau-5 (residues 330 to 558), which spans Tau-5* and Ct (Fig. 2C and fig. S2, E and F). We found that, although the CSPs induced by EPI-001 are small, they are highest for Tau-5* and allTau-5 (Fig. 2, D and E, and fig. S2, G and H), indicating that the main site of interaction of EPI-001 is in Tau-5* and, consequently, that the CSPs observed in Nt and Ct in the full-length AD are likely indirect.

The capacity of EPI-001 to increase AR AD oligomerization suggests that it directly interacts with an oligomeric form. To investigate this hypothesis, we generated a chimeric form of Tau-5* C404S by forming a disulfide bond between the side chains of the C-terminal Cys residues (C448) of two different Tau-5* C404S molecules, called 2Tau-5*, and compared its properties to those of 1Tau-5*, which we obtained by reacting with iodoacetamide the C448 side chain of Tau-5* C404S (Fig. 2F) (*29*). First, we used DLS to compare the oligomerization propensity of 1Tau-5* and 2Tau-5*. We found that, at the same mass concentration, 2Tau-5* had a higher propensity to form high-order oligomers than 1Tau-5* (Fig. 2G and fig. S2I) due to its increased valency (*30*). Next, we analyzed the chemical shift differences between 1Tau-5* and 2Tau-5* (fig. S2. J and K), which were equivalent to those induced by increasing the protein concentration of Tau-5* (*26*), in agreement with the DLS results. In addition, the ^13^Cα and ^13^C′ chemical shifts indicated that oligomerization increases the helicity of helix h2 (Fig. 2H), similarly to the results obtained with TDP-43 (*29*).

Our results indicate that introducing a covalent bond between C-terminal Cys residues increases the oligomerization propensity of Tau-5* C404S while preserving the site of interaction of EPI-001, providing us with an opportunity to test whether EPI-001 has a higher affinity for the oligomeric than for the monomeric form of its target. In agreement with this hypothesis, larger CSPs were observed in 2Tau-5* than in 1Tau-5* in the presence of EPI-001 (Fig. 2I and fig. S2, G, K, and L). Last, we used microscale thermophoresis (MST) to confirm the interaction between EPI-001 and 2Tau-5* by using an orthogonal biophysical technique (fig. S2M). We therefore concluded that EPI-001 preferably interacts with an oligomeric form of AR AD that is more structured than the monomer (Fig. 2H).

### EPI-001 modulates the network of interactions of the AR AD

**Fig. 3.**
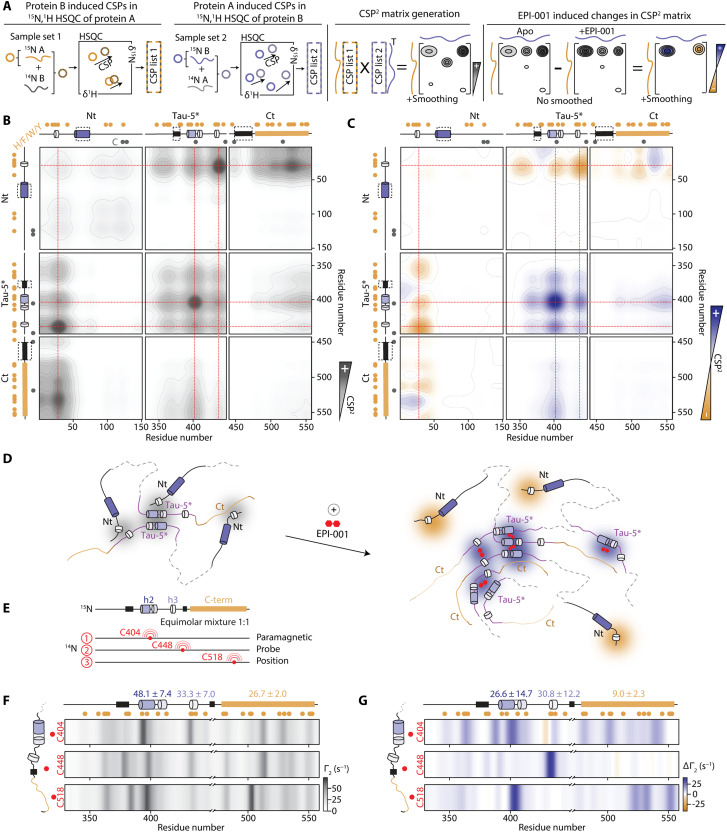
EPI-001 modulates the network of interactions within AR AD and strengthens homotypic interactions between residues in Tau-5*. (**A**) Schematic illustrating how CSP matrices are generated from reciprocal NMR titrations between AR AD fragments (examples: proteins A and B). CSPs measured in 2D ^1^H-^15^N NMR experiments of each labeled fragment upon addition of its unlabeled partner are combined to construct a symmetric CSP matrix that highlights regions involved in interactions. The schematic also shows how differences between CSP matrices measured in the absence and presence of EPI-001 are analyzed to reveal compound-induced changes in the interaction pattern. (**B**) CSP matrix for apo AR AD fragments, reporting regions that induce CSPs in each other. Interactions are detected between aromatic regions, with the highest CSPs observed in those aromatic segments that also contain partially helical motifs. Helical motifs enriched in aromatic residues are indicated by red dashed lines, and aromatic and Cys positions are marked by orange and gray circles, respectively. Fragment names are indicated above each panel, and dashed black outlines on the fragments schemes mark regions which were not assigned. (**C**) Difference CSP matrix highlighting changes upon addition of 1 molar equivalent of EPI-001. Blue indicates increased contacts, and orange indicates decreased contacts. EPI-001 enhances homotypic interactions within Tau-5*, particularly in aromatic and partially helical regions. (**D**) Schematic representing interactions between AR AD regions and the changes induced by EPI-001. (**E**) Experimental design of PRE experiments to monitor intermolecular interactions between allTau-5. (**F**) Γ_2_^HN^ rates representing the strength of intermolecular contact in the absence of EPI-001. (**G**) ΔΓ_2_^HN^ induced by adding 1 molar equivalent of EPI-001. (F and G) The average values of Γ_2_^HN^ for the h2, h3, and C-term regions are shown above the plots.

To investigate how the network of homo- and heterotypic interactions defining the conformational ensemble of the AR AD is modified by EPI-001, we first measured the CSPs induced by constructs Nt, Tau-5*, and Ct on the resonances of the same constructs in equimolar solutions (fig. S3A). To summarize the information contained in these nine experiments, we computed for pairs of residues a contact parameter, CSP^2^*_ij_*, as the product of the CSPs measured for residues *i* and *j* in the two reciprocal experiments (Fig. 3A), obtaining the CSP matrix shown in Fig. 3B; for consistency, CSP^2^_*ii*, apo_ was computed as the square of the CSP observed for residue *i*. We found that pairs of residues in motifs ^23^FQNLF^27^ and ^433^WHTLF^437^ have overall high CSP^2^_apo_ values, suggestive of a direct interaction between these motifs, and a similar result was obtained for the former motif and regions rich in aromatic residues in Ct and Tau-5*, although with lower values (Fig. 3B and fig. S3A). To validate that high values of CSP^2^_apo_ reflect the network of interactions, we used paramagnetic relaxation enhancement (PRE) NMR experiments. Specifically, we used these to measure intra- and intermolecular interactions involving C404 in the full-length AR AD, as this native Cys residue is found in the site of interaction of EPI-001 (fig. S3B).

The CSP matrix also reveals the relative oligomerization propensity of the fragments: Nt and Ct have lower CSP^2^ values, indicating a lower propensity to oligomerize than Tau-5* (Fig. 3B and fig. S3A) (*26*). Given that Tau-5* and Ct have similar aromatic characters (fig. S2E), this result suggests that the conformational properties of a region of sequence, such as its propensity to form secondary structures (fig. S2F), influence its propensity to oligomerize. In line with this, the motifs with some of the highest CSP^2^ values have high helical propensity or fold into ɑ helices upon interaction to globular partners (fig. S2F) (*31*, *32*). To confirm this, we remeasured the CSP matrix after introducing three helix-breaking substitutions (L26P in Nt and A398P and L436P in Tau-5*) and observed lower and less well-defined CSP^2^ values, reflecting weaker, less specific interactions, and in line with the notion that aromatic residues in helices are particularly prone to be involved in interactions (fig. S3, C and D).

To characterize the population shifts caused by EPI-001 on the AR AD, we next analyzed how this small molecule influences the CSP matrix (Fig. 3A). We found that EPI-001 appeared to strengthen (ΔCSP^2^ > 0, blue) the homotypic interactions between the region of sequence centered around residue 400, partially helical and rich in aromatic residues, and instead weakened (ΔCSP^2^ < 0, orange) heterotypic interactions between the motif ^23^FQNLF^27^ and the rest of the sequence (Fig. 3, C and D, and fig. S3A). On the basis of this result, we hypothesized that EPI-001 competes with the ^23^FQNLF^27^ motif for interaction with the helical motifs h2 and h3 in Tau-5*. To test this, we used variant AR AD* (*15*), with a helix-breaking mutation (L26P) in motif ^23^FQNLF^27^. Since, as shown above (fig. S3, C and D), the helical character of regions or motifs rich in aromatic residues increases their propensity to engage in interactions, we reasoned that this mutation would decrease the interaction of motif ^23^FQNLF^27^ with Tau-5*, thus allowing EPI-001 to more effectively target this subdomain. In agreement with this hypothesis, EPI-001 induced higher CSPs in h2 and h3 of AR AD* than of AR AD (fig. S3, E and F).

Last, we measured intermolecular PREs in construct allTau-5 to validate that high values of ΔCSP^2^ are due to direct interactions induced by EPI-001: We produced constructs in which two of the three native Cys residues (C404, C448, and C518) were substituted by Ser, labeled the remaining Cys residue with a paramagnetic probe and measured its effect on the resonances of the allTau-5 enriched in ^15^N (Fig. 3E). In agreement with the CSP matrix, the regions of sequence of allTau-5 with aromatic and partially helical character interacted with one another (Fig. 3F), and addition of EPI-001 strengthened these interactions (Fig. 3G and fig. S3, G and H).

**Fig. 4.**
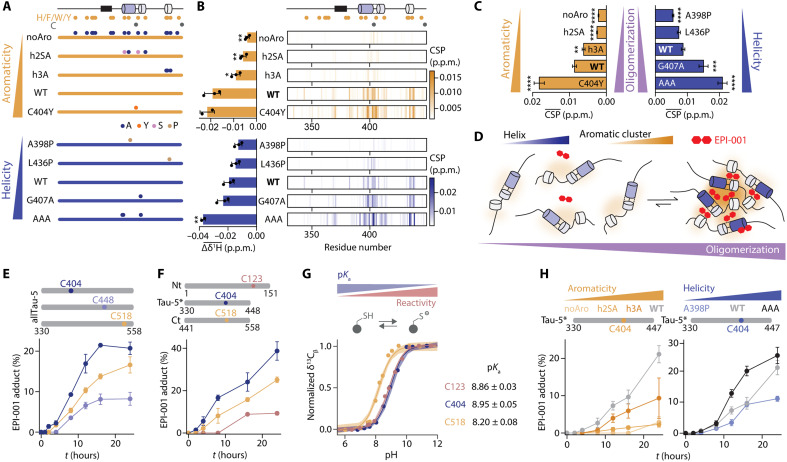
Tau-5* forms a transient EPI-001 interaction site upon oligomerization, induced by helical regions rich in aromatic residues. (**A**) Schematic of Tau-5* mutants. Orange and gray circles indicate the position of aromatic or Cys residues, respectively. (**B**) (Left) Average shifts of three (two for AAA and C404Y, due to line broadening) EPI-001 ^1^H signals induced by 1 molar equivalent of the corresponding Tau-5* mutant. (Right) Per-residue CSPs in the 2D ^1^H-^15^N NMR correlation spectra of Tau-5* upon addition of 1 molar equivalent of EPI-001. (**C**) Average protein CSPs induced by increasing the protein concentration from 25 to 400 μM in 2D ^1^H-^15^N NMR correlation spectra. Error bars are the SEM. (**D**) Scheme illustrating the preferential interaction of EPI-001 to the oligomeric species that contain a higher density of aromatic patches and helical elements. (**E**) Percentage of covalent adduct as a function of time using intact MS (fig. S4G). Each allTau-5 mutant contains a single Cys, while the remaining two were substituted to serines. (**F**) The percentage of covalent adduct formed in samples of AR AD fragments as a function of time using intact MS. Each AR AD fragment contains a single Cys. (**G**) Cys p*K*_a_ values of AR AD fragments quantified by monitoring the pH-induced shifts in the Cys ^13^C_β_ NMR signals. Errors indicate the standard error of p*K*_a_ estimation. (**H**) Percentage of covalent adduct, in sample of the Tau-5* mutants, as a function of time using intact MS. [(E), (F), and (H)] Error bars denote SDs (*n* = 3); in (H), the Tau-5* noAro measurement at 24 hours used *n* = 2.

Next, we focused on the sequence determinants of the selective interaction between EPI-001 and the oligomeric AR AD. Since the interaction site of EPI-001 has a high helical propensity and a high density of aromatic residues (fig. S2, E and F), we introduced amino acid substitutions in Tau-5* designed to investigate, independently, how these sequence features influence its interaction with EPI-001 (Fig. 4A). To alter helical propensity, we introduced helix-breaking substitutions in helices h2 and h3 (A398P and L436P) and helix-stabilizing substitutions in helix h2 (a single substitution, G407A, and a triple substitution, termed AAA, including substitutions G394A, S395A, and G407A) (Fig. 4A and fig. S4A). To alter aromatic character, we replaced all aromatic residues with Ala (noAro) or, to achieve the same effect locally, substituted by Ala or Ser those in helix h2 (h2SA variant: Y393S, W397A, Y406S, and H413A) or helix h3 (h3A variant: W433A, H434A, and F437A); we also replaced C404 with Tyr (Fig. 4A), thus increasing aromatic character. We found that noAro has a higher helical propensity than wild type (WT), as expected given the high helical propensity of Ala residues, and that substitution C404Y had a negligible effect on the secondary structure of this construct (fig. S4A).

These substitutions influenced the interaction of Tau-5* with EPI-001, observing a clear correlation between the aromatic and helical characters of the variants and the size of the CSPs caused by their interaction with EPI-001 (Fig. 4B and fig. S4B). To investigate whether aromatic and helical characters determine the ability of EPI-001 to interact with the AR AD because they are determinants of oligomerization, we next measured the CSPs induced by increases in protein concentration (fig. S4, C and D) in the absence of EPI-001. We obtained that Tau-5* oligomerization increases monotonically with aromatic character and helical propensity (Fig. 4C and fig. S4E), indicating that oligomeric Tau-5* is stabilized by interactions between aromatic side chains in regions of sequence rich in secondary structure, as recently described for the IDR of eIF4B (*33*) and that the selective interaction between EPI-001 and Tau-5* relies on the oligomerization of its target (Fig. 4D).

It has been proposed that EPI-001 is a covalent AR inhibitor that reacts with nucleophilic side chains of Cys residues in the AR AD (fig. S4F) (*11*). To determine whether its interaction with the target influences the rate of covalent attachment to Cys residues, we measured the reaction rates of three variants of the allTau-5 construct, which contains three such residues (C404, C448, and C518) by using mass spectrometry (MS; fig. S4G). In each variant, two Cys residues were mutated to Ser, leaving a single Cys available for reacting with EPI-001. We observed that the reaction was markedly faster with C404 (Fig. 4E), located in Tau-5* region, which harbors the site of interaction of EPI-001. To further confirm that C404 exhibits the highest reactivity, we examined the covalent modification rate of AR AD fragments—Nt, Tau-5*, and Ct—each containing only one Cys residue (C123, C404, or C518, respectively). Additional Cys residues were either mutated to Ser (C129S in Nt) or deleted (C448 in Tau-5* and Ct). Consistent with the results obtained for allTau-5, C404 reacted faster (Fig. 4F). This difference in reactivity is not due to differences of nucleophilic character as determined indirectly by measuring the p*K*_a_ (where *K*_a_ is the acid dissociation constant) of the thiol group of the difference Cys residues (Fig. 4G) (*34*–*36*).

Last, we investigated how the reactivity of C404 is influenced by the strength of the reversible interaction between EPI-001 and Tau-5*. We found that reactivity correlates directly with the helical and aromatic character of the construct (Fig. 4H), suggesting that the enhanced reactivity of C404 results from the prolonged residence time of EPI-001 at its interaction site in Tau-5*. In addition, since the detection by MS of the adduct provides direct evidence for the reaction between Tau-5* and EPI-001, these results confirm that CSPs reliably report on the strength of the reversible interaction between these EPI-001 and its target.

### Effects of EPI-001 partitioning in NR AD condensates

Several independent studies have established that the activity of AR as a transcription factor relies on its capacity to condense upon interaction with DNA (*15*, *37*–*39*), and thus phenotypic screens based on the high-throughput analysis of AR condensation dynamics, rather than on the measurement of transcriptional activity, can be used to identify AR inhibitors (*37*). More generally, since biomolecular condensates represent liquid phases distinct from the solutions surrounding them, it has been suggested that small molecules may selectively partition in them without necessarily engaging in direct interactions, a process that could facilitate target engagement and therefore be exploited for drug discovery and development (*40*, *41*). This concept has recently been tested both in vitro and in cells, revealing that the physicochemical properties of condensates are related to those of the small molecules that partition in them (*42*, *43*).

**Fig. 5.**
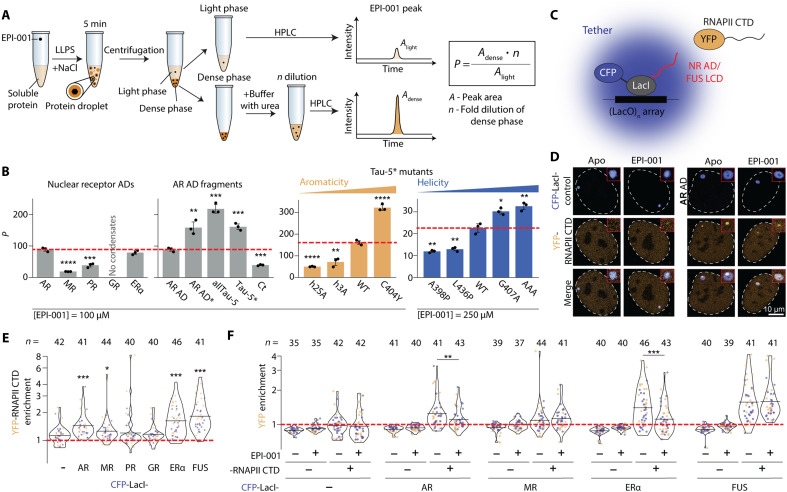
EPI-001 partitioning reduces the ability of the NR condensates to recruit RNAPII CTD in live cells. (**A**) Scheme of the EPI-001 partition coefficient experiment, where LLPS refers to liquid-liquid phase separation. (**B**) EPI-001 partition coefficients into condensates of NR ADs, AR AD fragments, and Tau-5* mutants. Condensate formation was induced with 1.75 M NaCl for NR ADs, AR AD fragments, and Tau-5* mutants that modulate aromaticity; for Tau-5* mutants that modulate helicity, 0.5 M NaCl was used. *P* was also determined for NR AD condensates at 500 mM NaCl (fig. S5E). EPI-001 concentrations were adjusted within solubility limits at different ionic strengths. The measured partition coefficient reflects both direct interactions and general physicochemical partitioning of the small molecule, which is sensitive to ionic strength. (**C**) Illustration of the condensate tethering experiment. (**D**) Exemplary images of cells cotransfected with YFP-RNAPII CTD and CFP-LacI-AR AD expression vectors. Cells were treated either in the absence (apo) or presence of 25 μM EPI-001. Each image depicts a single nucleus, delineated by a white dashed line. Within the red squares, a zoomed-in version of the CFP focus is displayed. Notably, the RNAPII CTD signal does not entirely overlap with the tether, consistent with known characteristics of RNAPII CTD recruitment in this assay (*45*, *46*). (**E**) Quantitative analysis of YFP-RNAPII CTD enrichment in NR AD and FUS LCD tether foci. Each data point represents a single tether/cell, with the number of tethers acquired for each condition indicated above the plot. Data are compiled from two independent transfections (orange and blue). (**F**) Quantification of YFP (−) or YFP-RNAPII CTD (+) enrichment in various tethers. Enrichment levels of RNAPII CTD in DMSO (−) and EPI-001 (+)—treated samples were normalized to RNAPII CTD cotransfected with empty LacI-CFP under corresponding conditions. Data originate from two independent transfections, distinguished by color (orange and blue).

The aromatic character of the AR AD is key for condensation likely due to the ability of aromatic residues to engage in π-π interactions (*15*). These interactions can be homotypic, with the ADs of other AR molecules, or heterotypic, with other components of transcriptional condensates such as RNAPII which, like the AR AD, is enriched in Tyr residues in its C-terminal domain (CTD), that is intrinsically disordered (*15*, *37*–*39*). In addition, EPI-001 partitions in the condensates formed by the AR AD, and modifying the structure of this small molecule to increase its affinity for the AR AD, as well as its partitioning in AR AD condensates, enhances its potency as an AR inhibitor (*15*). To determine whether selective partitioning contributes to selectivity, we first measured the partition coefficient of EPI-001 in the condensates (Fig. 5A) formed by the selectivity panel in vitro.

We found that the GR AD does not form droplets in vitro (fig. S5, A to C) and that EPI-001, bearing two aromatic rings, hardly partitions in PR and MR ADs droplets (Fig. 5B and fig. S5, D and E). By contrast, EPI-001 partitions similarly in the condensates formed by the ERα AD, which is the AD most enriched in aromatic residues, as well as those formed by the AR AD that has a lower aromatic character (Fig. 1C) but harbors the interaction site of EPI-001 (Fig. 5B). These results indicate that small molecules bearing aromatic rings partition in condensates formed by sequences rich in aromatic residues, in line with recent findings (*15*, *42*, *43*), and that the partitioning coefficient reflects both general physicochemical partitioning and direct interactions.

We next studied whether EPI-001 partitioning into NR AD droplets in vitro correlates with the behavior in cells. For this, we used a cell-based condensate tethering system, in which the NR ADs are expressed as a fusion protein with the DNA binding domain of the Lac repressor (LacI) and cyan fluorescence protein (CFP) in cells containing an integrated array of LacO binding sites (CFP-LacI-IDP) (*44*). In this experimental setup, the enrichment of a second, fluorescently tagged protein can be visualized and quantified in the tethered condensate (Fig. 5C) (*39*, *45*–*47*). We measured the recruitment of RNAPII CTD, a key client protein in transcriptional condensates (*48*), as a proxy of copartitioning driven by aromatic residues (*15*, *49*, *50*). RNAPII CTD was fused with yellow fluorescence protein (YFP) into lacO-induced condensates formed by the NR ADs in the condensate tethering system (Fig. 5D and fig. S5, F to H). The FUS low-complexity domain (LCD) was included as a positive control, as RNAPII CTD is known to partition into FUS LCD condensates (*51*, *52*). RNAPII CTD was significantly enriched in condensates formed by AR and ERα ADs and to a lesser extent in those formed by MR AD but showed no significant enrichment in GR and PR AD condensates (Fig. 5E and fig. S5, F and H). Then, to test the effect of EPI-001 on the recruitment of RNAPII CTD into condensates, we measured RNAPII CTD levels in the presence of the compound for the condensates that were enriched with RNAPII CTD: AR AD, MR AD, ERα AD, and FUS LCD. In good agreement with in vitro results (Fig. 5B), we observed a significant reduction in the enrichment of RNAPII CTD in both AR AD and ERα AD condensates upon treatment with the small molecule, but not in MR AD and FUS LCD condensates (Fig. 5F and fig. S5, F to H). These findings indicate that EPI-001 partitions into transcriptional condensates and that interactions between aromatic rings remain relevant in a cellular environment.

We next investigated the role of direct interactions in partitioning. For this, we measured the partition coefficient of EPI-001 in the droplets formed by AR AD fragments and variants that interact with this small molecule to different extents (Fig. 2D and fig. S2G). First, we used constructs allTau-5, Tau-5*, and Ct (Fig. 5B and fig. S5, C and D). EPI-001 partitioned better in those containing the interaction site of EPI-001, namely, Tau-5* and allTau-5, than in Ct, despite it being rich in aromatic residues (Fig. 5B and fig. S2E). Also, EPI-001 partitioned better in Tau-5* and allTau-5 than in full-length AR AD, which has the motif ^23^FQNLF^27^: Since this motif competes with EPI-001 for Tau-5* (fig. S3, E and F), the partition coefficient of this small molecule is higher in AR AD* droplets, where a helix-breaking substitution (L26P) weakens the interaction between ^23^FQNLF^27^ and Tau-5*. We also measured the partition coefficient of EPI-001 in Tau-5* variants (Fig. 5B and fig. S5, C and D): We found that substitutions in Tau-5* that reduce the interaction between EPI-001 and Tau-5* and, therefore, the reactivity of C404—by decreasing the aromatic character or helical propensity of the site of interaction—decreased EPI-001 partitioning (Fig. 5B). Collectively, our results indicate that the interaction between EPI-001 and the AR AD has a strong influence on its partitioning in the AR AD droplets.

**Fig. 6.**
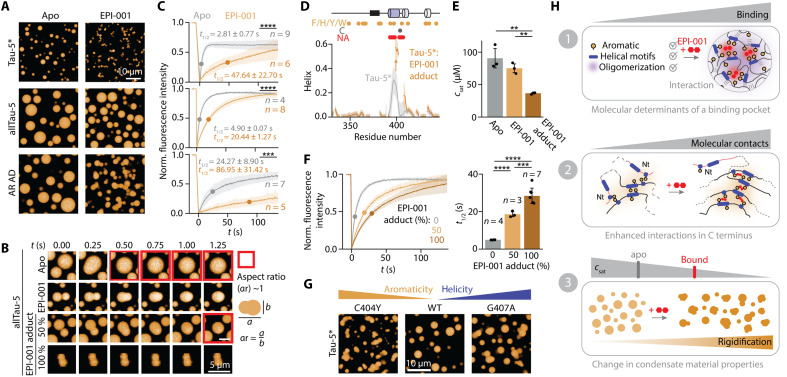
EPI-001 modulates AR AD condensates and induces their rigidification. (**A**) Fluorescence microscopy images showing in vitro reconstituted condensates formed by the AR AD constructs that contain the interaction site (Tau-5*, allTau-5, and AR AD) both in the absence and presence of EPI-001. (**B**) Fusion events monitored at different time points of allTau-5 droplets. Both the reversible interaction and covalent adduct forms of EPI-001 were measured. Spherical fused droplets are highlighted in red. (**C**) FRAP experiment of the AR AD fragment droplets. (**D**) NMR-derived helical content of Tau-5* and Tau-5*:EPI-001 adduct, using the δ2D algorithm (*71*). Orange and gray circles indicate the positions of aromatic and Cys residues, respectively. Red circles indicate not assigned residues due to line broadening. (**E**) Saturation concentration (*c*_sat_) measurements of allTau-5 in the presence and absence of EPI-001 (ratio, 1:1) and allTau-5:EPI-001 adduct (100%). Reduced *c*_sat_ indicates an enhancement of the condensation by the ligand. (**F**) FRAP experiments of allTau-5 condensates at different percentages of covalent adduct. (**G**) Fluorescence microscopy images of Tau-5* mutants. (**H**) (1) Schematic illustration depicting the molecular basis of the transient interaction site formed by AR AD molecules. (2) Diagram showing how EPI-001 modulates the network of interactions of AR AD, leading to increased oligomerization. (3) The drug effect on AR AD condensates: enhanced phase separation and rigidified condensates. [(E) and (F)] Error bars indicate SDs (*n* ≥ 3).

Last, to study how EPI-001 partitioning influences the properties of the AR AD droplets in vitro, we used three different constructs bearing the interaction site of this small molecule: AR AD, allTau-5, and Tau-5* (Fig. 2C). In all cases, we found that EPI-001 partitioning increased the number of arrested fusion events (Fig. 6, A and B, and fig. S6A), indicating a reduced dynamic character of the droplets, which we confirmed by using fluorescence recovery after photobleaching (FRAP) experiments (Fig. 6C). Similar to its effect in solution, where EPI-001 strengthens the homotypic interactions driving oligomerization, in the condensates, EPI-001 enhances the intermolecular homotypic interactions driving condensation, and as a result, the AR AD droplets exhibit a reduced dynamic behavior (*53*).

Since EPI-001 may inhibit the AR in part by reacting with Cys residues in the AR AD, we also analyzed how the covalent attachment of EPI-001 influences the properties of the target and the droplets that it forms (Fig. 6B). In solution, we found that this modification increases the helical propensity of the target (Fig. 6D and fig. S6B), likely by increasing its oligomerization propensity. In addition, since EPI-001 attachment increases the valency of the protein by substituting a Cys side chain by a moiety bearing two aromatic rings, it decreases the saturation concentration (*c*_sat_), cloud temperature (*T*_c_) (Fig. 6E and fig. S6C), and dynamic character of the droplets as monitored by FRAP experiments (Fig. 6F).

To dissect the individual contributions of increased helicity and aromaticity to the observed reduction in droplet dynamics, we generated two-point mutants: G407A, which increases the helical character of h2 without affecting aromatic content, and C404Y, which introduces an aromatic residue at the site of covalent modification. Both mutations resulted in the arrested dynamics of the protein condensates (Fig. 6G and fig. S6D). These data confirm that covalent modification reduces condensate dynamics by altering local sequence features—such as increasing aromaticity and structural order—which in turn stabilizes intermolecular interactions in the condensate.

## DISCUSSION

Unlike globular domains, whose well-defined structures can have pockets for interaction with small molecules, IDRs exhibit extreme conformational flexibility and dynamic interactions, making traditional drug-targeting strategies ineffective (*2*). We investigated the molecular basis of the selective interaction between EPI-001, a small molecule targeting an IDR that reached clinical trials (*11*, *12*), and its target, the AR AD: We reasoned that understanding in detail how these molecules recognize each other would suggest strategies to selectively target IDRs with small molecules.

Our results reveal that EPI-001 selectively stabilizes an oligomeric form of the AR AD that is more structured than monomeric AR AD. Oligomerization is driven by interactions involving aromatic residues in regions with helical propensity in the monomer (Fig. 6H), which are specific to the sequence of the AR AD and can lead to the formation of a relatively well-defined interaction site for this small molecule. Although we cannot definitely rule out that the shift in oligomerization equilibrium is due to a change in solvent quality, we favor a model where it is instead due to a direct and selective interaction between EPI-001 and oligomeric AR AD: This model provides a rationale for why amino acid substitutions that modify oligomerization propensity and, thus, the extent of direct interaction between these two molecules also change the rate of covalent attachment of EPI-001. Our work therefore provides a rationale for how a small molecule can interact selectively with an intrinsically disordered target and suggests that targeting IDRs with small-molecule drugs may be possible when the disordered target transiently adopts a druggable conformation upon oligomerization.

This mechanism is conceptually related to the exploitation of cryptic (transient) binding pockets for drug discovery against globular targets that appear to be undruggable due to their lack of binding pockets in structural representations based on a single conformation but which can be observed in molecular dynamic trajectories (*54*). The mechanism is particularly relevant for transcription factors, as increasing evidence suggests that the propensity of their IDRs to self-assemble, leading to the formation of transcription factor clusters or transcriptional condensates, plays a key role in their biological activity (*18*).

IDRs are defined by shallow energy landscapes displaying multiple minima (*2*, *55*). As a consequence of this, their conformational ensembles are highly sensitive to perturbations such as mutations and intermolecular interactions (*10*). In the case that we have studied, the interaction between EPI-001 and the AR AD strengthens the homotypic interactions that drive oligomerization and weakens heterotypic interactions such as that occurring between motif ^23^FQNLF^27^ and the region known as Tau-5, altering the physical properties of its phase-separated (Fig. 6H) state and ultimately reducing its transcriptional activity.

The transient nature of oligomerization limits the affinity that small molecules can have for intrinsically disordered targets, and our experiments as well as those reported for other similar systems (*8*) indicate that the affinities are in the millimolar range, which are likely insufficient for high potency in cells and in vivo. The selective interaction between small molecules and oligomeric IDRs can nevertheless be exploited for the development of covalent inhibitors and for proximity-based modalities (*56*), which do not require strong affinities and therefore appear as promising avenues for drug discovery for this highly challenging class of targets.

Last, our study also highlights the importance of both physicochemical properties (*42*, *43*) and direct interactions in rationalizing the partitioning of small molecules in biomolecular condensates: EPI-001 selectively partitions into condensates formed by the AR AD due to its aromatic character as well as to specific molecular interactions with the target. These results indicate that optimizing both the physicochemical properties of small molecules and their interactions with their targets will be important in the discovery of drugs targeting proteins forming biomolecular condensates, as we have recently shown for the AR AD (*15*).

In summary, our study shows that IDR oligomerization can provide opportunities for selectively targeting this challenging class of targets with small molecule drugs. The insights gained here pave the way for rational design strategies aimed at developing therapeutics against transcription factors and other IDR-containing proteins. In addition, they emphasize the importance of considering condensation and oligomerization as key factors in the development of inhibitors for intrinsically disordered targets.

## MATERIALS AND METHODS

### Sequence analysis

NR protein sequences [UniProt IDs: P10275 (AR), P08235 (MR), P06401 (PR), P04150 (GR), and P03372 (ERα)] were used to calculate the sequence identity for the ADs and the folded domains, and the conserved N-terminal Cys of the DBD (C-V/A-I/V-C motif) was arbitrarily set to classify between the AD and the DBD-LBD. In this study, ERα AD was taken as prototypical of the ER. As control, the sequence properties were also calculated for the IDR regions of TDP-43 (residues 262 to 414, UniProt ID: Q13148), FUS (residues 1 to 270, UniProt ID: P35637), EWS LCD (residues 1 to 264, UniProt ID: B1PRL2), Nup98 (residues 1 to 741, UniProt ID: P52948), hnRNPA1 (residues 186 to 372, UniProt ID: P09651), and CPEB4 (residues 1 to 448, UniProt ID: Q17RY0).

The sequence properties of the intrinsically disordered NR ADs were calculated using the CIDER package (*21*). The calculated parameters were the mean hydropathy using the Kyte-Doolittle scale (*57*); fraction of disorder promoting residues in the sequence; κ and Ω values describe the segregation of charged and proline residues as previously described (*58*, *59*); fraction of charged residue; and the net charge per residue. The aromatic clustering (Ω_aro_) was calculated by making a sequence length correction of a previously proposed parameter (*60*). This metric computes the average inverse distance among aromatic residues and normalizes it by the sequence length by calculating the Ω_aro_ value for a maximum clustered sequence (Ω_aro,clust._) and an ideally spaced aromatic sequence (Ω_aro, ideal._)

$$\Omega_{\text{aro},\text{norm}}=\frac{\Omega_{\text{aro}}-\Omega_{\text{aro},\text{ideal}}}{\Omega_{\text{aro},\text{clust}}-\Omega_{\text{aro},\text{ideal}}}$$

The sequence patterning plots shown in fig. S1B were calculated by measuring a rolling mean average of 30 residue windows. The hydropathy plot took into account the Kyte-Doolittle scale (*57*). For the charge plot, Arg and Lys residues had a +1 value and aspartate and glutamate residues a −1 value at pH 7.4.

To quantify the sequence patterning, we calculated *z*-scores using the NARDINI program (*19*, *61*). The *z*-score reflects on the degree of blockiness of groups of residues compared to 10^6^ randomly generated sequences with the same composition. Residues are grouped into the following eight types: polar ≡ (Q,S,H,T,C,N), hydrophobic ≡ (I,L,M,V), positive ≡ (K,R), negative ≡ (D,E), aromatic ≡ (F,Y,W), alanine ≡ A, proline ≡ P, and glycine ≡ G. Considering all pairs of residue types leads to 36 patterning features, where 6 correspond to Ω parameters—same type segregation—whereas the other 30 are the patterning of different class comparisons or δ-parameter. The positive *z*-scores imply that the patterning of the two residue types is more blocky than random, whereas negative *z*-scores imply that the patterning is better-mixed. To visualize the disordered region of the AR AD (Fig. 1A), we used the DODO package (*62*); source code is available as an additional resource at https://github.com/ryanemenecker/dodo.

### Protein sequences

The sequences of proteins used for the in vitro experiments are provided in table S1.

### Protein expression

Recombinant expression of nonisotopically labeled proteins was produced in lysogeny broth (LB), whereas isotopically labeled (^15^N or double-labeled ^15^N and ^13^C) proteins were expressed in minimal M9 media supplemented with ^15^NH_4_Cl and, if needed, ^13^C-glucose. Cell cultures at optical density at 600 nm of 0.6 were induced with 1 mM isopropyl-β-d-thiogalactopyranoside (IPTG) and incubated under specific conditions for expression.

Expression trials were performed for most of the protein constructs used in this study. Depending on the protein, different expression vectors, bacterial cell strains, or expression conditions were chosen. The different AD of the NR family: AR (1 to 558 amino acids, with 21Q and 24G polyQ and polyG tracts at positions 58 to 78 and 449 to 472, respectively), AR* (L26P) (*15*), AR C404 (all Cys deleted, except C404), MR (1 to 602 amino acids), PR (1 to 566 amino acids), GR (1 to 420 amino acids), ERα (1 to 184 amino acids); Ct constructs (441 to 558 amino acids): Ct, Ct 4G (containing 4G in polyG tract), Ct C518 (containing 4G in polyG tract and the C448 is deleted); and Tau-5* constructs (330 to 448 amino acids): Tau-5* and its mutants—A398P + L436P, C404Y, A398P, L436P, G407A, C404S, and C404 (lacking the last residue C448)—were recombinantly produced in *Escherichia coli* Rosetta (DE3) cells. Upon induction with IPTG, the expression was carried out in LB or M9 media at 25°C overnight under agitation. noAro (where all the aromatic residues were substituted by Ala), AAA (G394A + S395A + G407A), A398P + T435P, and T435P mutants of Tau-5* were produced in *E. coli* BL21 (DE3) cells (New England Biolabs, #C2527) at 25°C overnight in both LB and M9 media. While h2SA (Y393S + W397A + Y406S + H413A) and h3A (W433A + H434A + F437A) mutants of Tau-5* were produced in *E. coli* BL21 (DE3) LysY (New England Biolabs, #C3010I) cells at 25°C overnight for unlabeled LB media and 37°C for 3 hours for ^15^N/^13^C M9 media. In all cases, the protein constructs were cloned in pDEST17 vector (Thermo Fisher Scientific).

The allTau-5 construct [330 to 558 amino acids, polyG with 4G, to prevent protein aggregation (*63*)] and allTau-5 24G (polyG with 24G), allTau-5 mutants CtoS (C404S + C448S + C518S), CtoS PP (C404S + C448S + C518S + A398P + L436P), and mutants containing only one Cys C404, C448, or C518 (with the other two Cys mutated to Ser) were produced in *E. coli* BL21 (DE3) LysY cells (New England Biolabs, #C3010I) transformed with pET-30a(+) plasmids (Novagen) at 25°C overnight in LB and M9 media.

For nonisotopically labeled Nt constructs (1 to 151 amino acids): Nt, Nt L26P, and Nt C123 (with C129S substitution), *E. coli* BL21 (DE3) LysY cells (New England Biolabs, #C3010I) were transformed with a pET-50b(+) plasmid (Novagen) with the solubility tag of NusA preceding the protein of interest cloned. The protein was expressed overnight at 37°C upon induction with IPTG. Isotopically labeled Nt C123 was produced under the same conditions but expressed at 25°C. Isotopically labeled Nt and Nt L26P were produced in *E. coli* Rosetta (DE3) and BL21 (DE3) cells, respectively, transformed with pDEST17 vector (Thermo Fisher Scientific) containing maltose binding protein (MBP) tag and expressed in M9 media overnight at 25°C. The use of solubility tags such as NusA and MBP prevented Nt aggregation (*27*). Notably, the pDEST17 vector with the MBP tag displayed higher yields in protein production in M9 media compared to pET-50b(+) with NusA. Every plasmid was engineered to contain a His-tag following the solubility tag (for Nt constructs) and either a His-3C or a TEV (Ct and Nt variants) cleavage site upstream of the encoded protein sequences.

### Protein purification

The purification was performed as previously reported (*15*, *26*, *27*). Briefly, for all protein constructs except Nt, cells were harvested by centrifugation and resuspended in phosphate-buffered saline (PBS). Then, the cells underwent two rounds of sonication for 7 min each with a 5-s on-and-off pulse. Following centrifugation, the supernatants were discarded, and the pellets were washed twice using a wash buffer [PBS, 1% Triton, 500 mM NaCl, and 1 mM dithiothreitol (DTT) (pH 7.8)]. A total of 5 mM MgSO_4_ and 130 μM CaCl_2_ were added in the first wash. Insoluble inclusion bodies were collected via centrifugation and subsequently solubilized overnight at room temperature in a binding buffer [20 mM tris, 500 mM NaCl, 5 mM imidazole, 8 M urea, 0.05% (w/v) NaN_3_, and 1 mM DTT (pH 7.8)]. The solubilized inclusion bodies underwent centrifugation and filtration of the supernatant, were applied to a HisTrap HP column (Cytiva) at room temperature, and eluted with a gradient of 500 mM imidazole in the binding buffer. Eluted fractions underwent two rounds of dialysis, of 3 and 16 hours, using a buffer [50 mM tris and 1 mM DTT (pH 8.0)]. His-tag cleavage was performed using either TEV or 3C proteases during the second dialysis step. The addition of 0.5 mM EDTA in the dialysis buffer for TEV protease was needed. After dialysis, urea was added to the sample to reach a final concentration of 8 M, and a second HisTrap HP column (Cytiva) was performed at room temperature to collect the cleaved protein in the flow-through. Concentration was achieved using 3- or 10-kDa Amicon concentrators (Merck) depending on the molecular weight of the respective protein. The samples were aliquoted and stored at −80°C. Notably, the allTau-5 and Ct constructs were better purified by adjusting the urea concentration to 6 M and adding 2 M GuHCl in the binding buffer.

For the Nt constructs, cells were harvested via centrifugation and resuspended in a core buffer [20 mM NaH_2_PO_4_, 500 mM NaCl, 5% (v/v) glycerol, 0.05% (w/v) NaN_3_, and 1 mM DTT (pH 8)]. Lysozyme powder was added to facilitate cell lysis at a concentration of 1.5 mg/ml, followed by incubation for 1 hour at 4°C on a spinning wheel. The lysed cells were then subjected to sonication for 20 min with a pulse of 5 s on and 10 s off. Upon centrifugation, the supernatant was filtered and loaded onto a HisTrap HP (Cytiva) column at 4°C. Fractions containing the protein of interest were eluted from the column using a gradient of 500 mM imidazole in the core buffer. The eluted fractions were concentrated using 10-kDa Amicon concentrators (Merck) and injected on a size exclusion chromatography (SEC) using a HiLoad Superdex 200 pg 26/600 column (Cytiva). The purest fractions were collected and mixed with either TEV or 3C protease. Subsequently, dialysis was conducted in a core dialysis buffer [50 mM NaH_2_PO_4_, 100 mM NaCl, and 1 mM DTT (pH 8), with the addition of 0.5 mM EDTA for TEV] at 4°C overnight. After dialysis, urea was added to the sample to reach a final concentration of 8 M, and a second HisTrap HP column (Cytiva) was performed at room temperature to isolate the cleaved protein which is present in the flow-through. This protein was concentrated using 3-kDa concentrators, aliquoted, and stored at −80°C.

### Small-molecule preparation

EPI-001 was purchased from Sigma-Aldrich (SML1844) and used in the experiments.

### Protein sample preparation

Stored protein aliquots at −80°C were thawed and subjected to SEC using HiLoad Superdex 75 or 200 pg columns (Cytiva) in a final buffer [20 mM sodium phosphate, 1 mM tris(2-carboxyethyl)phosphine (TCEP), and 0.05% (w/v) NaN_3_ at pH 7.4], maintained at 4°C unless specified otherwise. After the SEC, the fractions containing the pure protein were concentrated using either a 3- or 10-kDa Amicon concentrator (Merck) depending on the protein construct size. Subsequently, samples underwent centrifugation to remove aggregate traces, and their concentrations were determined using a microvolume spectrophotometer (NanoDrop, Thermo Fisher Scientific) at 280 nm, except for noAro samples, where determination via absorbance was not feasible due to the absence of aromatic residues, thus requiring the use of a high-performance liquid chromatography (HPLC) system. The absolute concentration of an noAro sample was initially determined using amino acid analysis. Subsequently, various dilutions were prepared to build a calibration curve using an HPLC system. This calibration curve was constructed by measuring the protein peak integration, detected at ~49% of ACN:H_2_O (9:1), across different concentrations. This provided a direct relationship between the protein concentration and the corresponding peak integral. The final samples were prepared on ice.

The samples involving the small-molecule EPI-001, including their apo versions, contained 0.5% dimethyl sulfoxide (DMSO) unless otherwise specified. EPI-001 was added from 50 or 100 mM DMSO or DMSO-*d*_6_ (for NMR experiments) stocks.

### Spin labeling for PRE measurement

Purified allTau-5 Cys-mutants—C404, C448, and C518—and AR C404 were reduced by incubating with 5 mM DTT, after which the solution was buffer-exchanged into a TCEP-free final buffer (see the “Protein sample preparation” section) by passing the protein solution through a size exclusion column HiLoad Superdex 75 or 200 pg 13/300 (Cytiva). Proteins were then incubated with 5 molar equivalents of S-(1-oxyl-2,2,5,5-tetramethyl-2,5-dihydro-1H-pyrrol-3-yl)methyl methanesulfonothioate (MTSL) (Toronto Research Chemicals), which was added from a DMSO stock, and allowed to react overnight with agitation at 37°C and pH 8. Unreacted MTSL and disulfide-bridged protein dimers were removed by SEC using a HiLoad Superdex 75 or 200 pg 16/600 column (Cytiva). Nearly 100% Cys modification was confirmed using intact MS.

### Cysteine thiol group blocking by iodoacetamide

Iodoacetamide (Merck) DMSO stocks, freshly prepared at 1 M, were used to block Cys of Tau-5* C404S (to create 1Tau-5*) and AR AD C404. First, proteins were reduced by incubating with 5 mM DTT, after which the solution was buffer-exchanged into a TCEP-free final buffer (see the “Protein sample preparation” section) by passing the protein solution through a size exclusion column HiLoad Superdex 75 or 200 pg 13/300 (Cytiva). Then, protein samples were incubated with 10 molar equivalents of iodoacetamide, at pH 8 and 37°C for 1 hour. Unreacted iodoacetamide and disulfide-bridged protein dimers were removed by SEC using a HiLoad Superdex 75 or 200 pg 16/600 column (Cytiva) with the TCEP-free final buffer.

### Generation of 2Tau-5*

The Tau-5* C404S sample was buffer-exchanged into a TCEP-free final buffer at pH 8 (see the “Protein sample preparation” section) by passing the protein solution through a size exclusion column, HiLoad Superdex 75 13/300 (Cytiva). Some dimer formation via disulfide bridge between C448 residues (2Tau-5*) occurred on the column. Monomeric peak of Tau-5* C404S was collected and incubated at pH 8 and 37°C for 48 hours. Monomeric Tau-5* C404S and 2Tau-5* were then separated using a size exclusion column, HiLoad Superdex 75 16/600 (Cytiva), with the TCEP-free final buffer.

### Generation of the EPI-001 protein adduct

The allTau-5:EPI-001 adduct was prepared by incubating allTau-5 with EPI-001 at a 1:5 molar ratio in the final buffer at pH 8 and 37°C for 72 hours. Afterward, a SEC using a HiLoad Superdex 75 pg 26/600 column (Cytiva) was conducted to eliminate formed multimers and free EPI-001. The efficacy of the reaction was evaluated through intact MS (fig. S7, A and B). For ^15^N, ^13^C-double labeled Tau-5*:EPI-001 adduct, Tau-5* C404, which contains one Cys per molecule (i.e., C448 was deleted), was incubated with EPI-001 at a 1:10 molar ratio in the final buffer at pH 8 and 37°C for 96 hours. Separation of the adduct from the protein and free compound was achieved using a Jupiter C4 semiprep column (Phenomenex) connected to an Agilent Technologies 1200 HPLC instrument. Mobile phases consisting of H_2_O and ACN:H_2_O (9:1) with 0.1% (v/v) trifluoroacetic acid (TFA) were used. The elution of Tau-5*:EPI-001 occurred with 45% (v/v) ACN:H_2_O. The fraction containing the adduct was collected and lyophilized, followed by redissolution in the final buffer with the pH readjusted to 7.4.

Concentrations of both adducts were determined by ultraviolet (UV) absorbance at 280 nm using a NanoDrop spectrophotometer (Thermo Fisher Scientific). One representative preparation of each adduct was quantified by amino acid analysis to obtain an absolute concentration. Serial dilutions of this amino acid–quantified material were measured by NanoDrop to generate calibration curves (absorbance at 280 nm versus concentration). Concentrations of subsequent samples were calculated by interpolation from the corresponding calibration curve.

### Amino acid analysis for protein quantification

The protein samples were hydrolyzed in glass tubes with HCl 6 M [0.1 to 1% (v/v) phenol] for 24 hours at 110°C. The solvent was evaporated, and the remaining powder was redissolved in water and filtered. An aliquot of the filtered sample containing the amino acids (AA) was derivatized with 6-aminoquinolyl-*N*-hydroxysuccinimidyl carbamate (AQC) following the indication of the AccQ-Tag method (Waters) to obtain the corresponding AQC analogs (AQC-AA). The derivatized AQC-AA were injected in a Nova-Pak C18 (4 μm) HPLC column (Waters) connected to a Waters 600 HPLC system with a detector Waters 2487. The amount of AQC-AA was followed by measuring the absorbance at 254 nm. Concentration of amino acids was calculated by an internal standard method. A known amount of aminobutyric acid (AABA), as internal standard, was added to the sample, and the analyte amount was calculated using area responses of analytes and internal standard. Working amino acid standard solutions were prepared by dilution of commercial 2.5 mM stock (Amino Acids Mix Solution 79248, Sigma-Aldrich). Internal standard solutions (2.5 mM) are prepared using norleucine and AABA (Sigma-Aldrich).

### NMR experiments

All NMR experiments were recorded at 5°C using Bruker Ascend Evo 1 GHz, Bruker Avance Neo 800 MHz, and Bruker Avance III 600 MHz spectrometers equipped with TCI (Triple Resonance Cryoprobe Inverse) cryoprobes. Samples, unless otherwise specified, were measured in 3-mm NMR tubes containing the final buffer [20 mM sodium phosphate buffer (pH 7.4), 1 mM TCEP, and 0.05% (w/v) NaN_3_] 10 μM sodium 3-(trimethylsilyl)propane-1-sulfonate for chemical shift referencing, and 10% (v/v) D_2_O. All three-dimensional (3D) triple-resonance experiments were acquired with nonuniform sampling applied to both indirect dimensions.

Data processing encompassed qMDD (*64*) for nonuniformly sampled data reconstruction and NMRPipe (*65*). Subsequent analysis was carried out using CcpNMR Analysis (*66*).

### Assignments

Backbone assignment of ^15^N, ^13^C-double labeled ERα AD (150 μM) and GR AD (280 μM) was accomplished using the BEST-TROSY versions (*67*) of 3D HNCO, HNCA, HNCACB, and HNcoCACB experiments in combination with 3D HNcaCO and hNcaNNH experiments (*68*). For backbone assignment of PR AD, a 200 μM ^15^N,^13^C-double labeled sample prepared in the final buffer (pH 5.8) and 8% (v/v) D_2_O was used. BEST-TROSY versions of 3D HNCO, HNcaCO, HNCA, HNcoCA, HNCACB, and HNcoCACB, in combination with 3D HNcaCO and hNcocaNNH and 4D HNCACO, were recorded.

For MR AD, two 200-μM samples were used, one in the absence and the other in the presence of 2 M urea, containing 2% (v/v) D_2_O. For each sample, BEST-TROSY versions of 3D HNCO, HNCA, HNCACB, and HNcoCACB experiments in combination with 3D HNcaCO and hNcaNNH experiments were acquired.

Backbone assignments for Nt and Ct were previously reported [BMRB (Biological Magnetic Resonance Bank) IDs: 25607 and 51476, respectively] (*15*, *27*). For the ^15^N,^13^C-double labeled allTau-5, allTau-5 CtoS, and allTau-5 CtoS PP at 150 μM, assignments were accomplished through a series of BEST-TROSY versions of 3D experiments, including HNCO, HNCA, HNCACB, HNcoCACB, and HNcaCO. In addition, the 3D hNcaNNH experiment was measured for allTau-5. AllTau-5 24G assignments were derived from allTau-5 and confirmed using HNCA and HNCO experiments.

Backbone assignment of ^15^N,^13^C-double labeled Nt L26P at 150 μM was achieved using 3D HNCA, HNCO, HNcaCO, and HNCACB experiments. Assignments for 150 μM Tau-5* mutants (noAro, C404Y, A398P, G407A, and AAA), 12 μM Tau-5* C404 and Tau-5*:EPI-001 adduct, and 1Tau-5* and 2Tau-5* (0.31 mg/ml) were obtained through BEST-TROSY versions of 3D HNCO and HNCA experiments. Additional spectra recorded were HNcaCO for noAro, A398P, and AAA; HNCACB for noAro and AAA; and HNcoCACB for noAro, G407A, A398P, and Tau-5*:EPI-001 adduct. Assignments for h2SA and h3A were derived from Tau-5* and noAro assignments, while those for L436P, A398P + L436P, T435P, and A398P + T435P mutants of Tau-5* were established using assignments from Tau-5* and allTau-5 CtoS PP.

### Binding studies

To evaluate EPI-001 reversible interaction with protein constructs, CSPs were determined by combining ^1^H and ^15^N chemical shift changes

$$\text{CSP}=\sqrt{{\left(\Delta \delta^{1}H\right)}^{2}+{\left(\frac{\Delta \delta^{15}N}{5}\right)}^{2}}$$

Under the conditions used for NMR measurements (pH 7.4, 278 K, and 1 mM TCEP), no covalent binding of EPI-001 to Cys residues was detected (fig. S7C). For NR ADs at 25 μM, we used the 2D ^1^H,^15^N FHSQC pulse sequence with WATERGATE as a water suppression module (*69*) to assess CSPs in the absence and presence of 250 μM EPI-001. For other constructs, we used 2D ^1^H,^15^N BEST-TROSY. AR AD fragments (Nt, Tau-5*, Ct, and allTau-5 24G) and its mutant AR AD* (fig. S3E) were studied at a concentration of 25 μM. Tau-5* mutants were studied at 250 μM. Experiments were performed both in the absence and presence of EPI-001 (10 molar equivalents for AR AD fragments and 1 molar equivalent for Tau-5* mutants).

CSPs in the 2D ^1^H,^15^N BEST-TROSY spectra of 1Tau-5* and 2Tau-5* at a protein concentration of 0.31 mg/ml (25 μM 1Tau-5* and 12.5 μM 2Tau-5*) were measured in the presence of 250 μM EPI-001. For Tau-5* C404, CSPs induced by covalent binding with EPI-001 were examined at a protein concentration of 12 μM.

^1^H chemical shift changes of EPI-001 in the presence of the protein constructs were observed through 1D ^1^H experiments using WATERGATE as the water suppression block (*70*). Three to four distinct peaks of EPI-001 were monitored—two aromatics, a methylene, and a methyl group.

### MR AD competition with AR AD for EPI-001 binding

To assess competition between equimolar amounts of MR AD and AR AD for EPI-001 binding, two 2D ^1^H, ^15^N BEST-TROSY spectra of ^15^N-labeled AR AD (25 μM) plus 250 μM EPI-001 (10-fold molar excess) were compared: one in the absence and the other in the presence of ^14^N MR AD. A potential interaction between both proteins was ruled out by recording an additional 2D ^1^H, ^15^N BEST-TROSY spectra of AR AD (25 μM) in the presence of 1 molar equivalent of ^14^N MR AD.

### Helical content measurement

The helical content for AR AD and its fragments (Nt, Tau-5*, and Ct; fig. S2F) was quantified at 25 μM protein and at the assignment concentrations for the ADs of PR (200 μM), MR (200 μM), GR (280 μM), and ERα (150 μM) (fig. S1E). For allTau-5 24G and Tau-5* constructs (WT, noAro, C404Y, A398P, G407A, and AAA), the concentration was set at 150 μM, unless otherwise specified in the figures. Tau-5* and Tau-5*:EPI-001 adduct were measured at 12 μM, with Tau-5* featuring a single Cys at position C404 in both cases (Tau-5* C404). For 1Tau-5* and 2Tau-5*, it was obtained at 0.31 mg/ml.

^1^H, ^15^N, C′, and C_α_ chemical shifts obtained from 2D ^1^H, ^15^N correlation spectra and 3D HNCO and HNCA experiments were used as an input for the δ2D algorithm (*71*). Error estimates were included in all δ2D helicity plots, following the approach of Camilloni *et al.* (*71*). Uncertainties of ±2% per residue were applied when comparing the same residue type with the same number of chemical shifts available, where systematic errors cancel, whereas uncertainties of ±10% per residue were applied in cases with incomplete data or residue identity changes (e.g., at mutation sites).

### CSP matrix

Unlabeled (^14^N) and isotopically labeled (^15^N) forms of protein constructs were mixed at a 200 μM concentration each. These experiments were carried out in the presence or absence of 200 μM EPI-001, with all samples containing 0.5% DMSO-*d*_6_, including all the samples in the absence of the small molecule. CSPs induced by adding 1 molar equivalent of unlabeled fragments were measured in 2D ^1^H,^15^N BEST-TROSY spectra of the labeled constructs (^15^N-Nt, ^15^N-Tau-5*, ^15^N-Ct, etc.). Detected CSPs reported on intermolecular interactions between fragments (e.g., ^15^N-Nt regions interacting with ^14^N-Nt, ^14^N-Tau-5*, or ^14^N-Ct).

We measured for each pair of interacting protein constructs the reciprocal experiments, i.e., the binding observed (NMR active nuclei, ^15^N) from each different construct. For a set of reciprocal datasets, e.g., ^15^N-Nt + ^14^N-Tau-5* and ^15^N-Tau-5* + ^14^N-Nt, we multiplied the two strings of CSP values to generate a *m*·*n* matrix, where *m* and *n* denote the length of the protein construct (e.g., for Nt + Tau5*, *m*_Nt_ = 151 and *n*_Tau5*_ = 119). The result of the product of two sets of CSP lists was defined as the CSP^2^. The value of CSP^2^ for a residue *i* will increase if, in the reciprocal dataset, its CSP and the one corresponding to residue *j* (CSP^2^*_ij_*) are large. In IDPs, the dynamic nature facilitates promiscuous contacts, but to ensure that certain interactions are taking place, mutations in specific regions were introduced to validate the contacts calculated. Also, a direct measurement of PRE NMR experiments, dependent on intermolecular distances, was conducted. The CSP matrix was represented as a heatmap, and a Gaussian filter was applied to average out missing data and emphasize regions with the highest CSPs caused by interactions.

Separate CSP matrices were computed for constructs both in the presence and absence of EPI-001 (or mutated helical motifs). Subsequently, the difference between these matrices was calculated (ΔCSP^2^ = CSP^2^_EPI-001_ − CSP^2^_apo_ or ΔCSP^2^ = CSP^2^_no helix_ − CSP^2^_helix_). It is important to note that if any of the corresponding values in one of the matrices (apo or with EPI-001) was missing or zero, the resulting position in the final matrix was set to zero. All data analysis was conducted with Python.

To confirm that the changes in the CSP matrices (fig. S3, C and D) are caused by disruptions in potential helical structure formation and not due to altered hydrophobicity resulting from the L436P mutation, we also evaluated the effect of the T435P mutation on the oligomerization of Tau-5*, which additionally contains the A398P mutation (fig. S7D).

### Paramagnetic relaxation enhancement

Intramolecular and intermolecular PREs for AR AD C404 were assessed by measuring 2D ^1^H,^15^N BEST-TROSY spectra of paramagnetic (para) and diamagnetic (dia) samples. The intensity ratio *I*_para_/*I*_dia_ was then calculated to quantify PREs.

For intramolecular PRE measurements, a sample containing 20 μM ^15^N-labeled AR AD with a paramagnetic spin label (MTSL) at C404 was mixed with 80 μM AR AD, where C404 was covalently blocked with iodoacetamide. Using these sample composition potential interactions between spin-labeled ^15^N AR AD and ^14^N AR AD would not produce PREs, minimizing but not completely preventing detection of intermolecular PREs. To account for intermolecular contributions, intermolecular PREs were measured at the same total protein concentration using a sample containing 20 μM ^15^N-labeled AR AD with C404 blocked with iodoacetamide, 20 μM AR AD spin-labeled at C404, and 60 μM AR AD with C404 blocked with iodoacetamide. The diamagnetic state was achieved by adding 1.5 mM ascorbic acid for 24 hours at 4°C to reduce the paramagnetic spin label for allTau-5 and AR C404 samples.

Intermolecular contacts of allTau-5 were measured on equimolar (100 μM) mixtures of spin-labeled ^14^N Cys-mutant allTau-5 (C404, C448, or C518) and ^15^N-labeled allTau-5 CtoS. A total of 200 μM EPI-001 was added to monitor the effects of the small molecule. The ^1^H^N^ *R*_2_ relaxation measurements were performed at 800 MHz at 5°C. A total of six *T*_2_ relaxation times were used (1, 2, 5, 10, 20, and 60 ms). The ^1^H^N^ PREs were quantified by fitting the decay of signals to a single exponential function to obtain *R*_2para,*i*_ and *R*_2dia,*i*_ rates, from which the PRE contribution was calculated as Γ_2,*i*_ = *R*_2para,*i*_ − *R*_2dia,*i*_. Exponential curve fits were performed by using in-house written scripts in R. Uncertainties in *R*_2_ were computed as the standard errors of the fit. In Fig. 3 (F and G), a Gaussian filter was applied for visualization. The average values were calculated for the following regions: h2 (^391^LDYSAWAAAAAQ^403^), h3 (^433^WHTLF^437^), and the C-term aromatic-rich region (479 to 558). These values correspond either to the averaged *Γ*_2_ (in the absence of EPI-001) or to ΔΓ_2_ (*Γ*_2,EPI-001_ − *Γ*_2,apo_) to assess the effect of EPI-001.

In addition, we analyzed intermolecular PREs of allTau-5 by plotting *I*_para_/*I*_dia_ ratios in the presence and absence of EPI-001, using spectra acquired with the 1-ms relaxation delay (fig. S3G). Standard errors for *I*_para_/*I*_dia_ values were estimated by propagating the intensity ratios of the individual spectra.

### Oligomerization studies

Oligomerization of 2Tau-5* and 1Tau-5* was analyzed by measuring CSPs in 2D ^1^H,^15^N BEST-TROSY experiments at a concentration of 0.31 mg/ml (25 μM 1Tau-5* and 12.5 μM 2Tau-5*). The samples also contained 0.5% DMSO-*d*_6_ to ensure consistency, as the same spectra were used for the EPI-001 binding studies.

To investigate the oligomerization of Tau-5* and its mutants, designed to modify aromaticity (noAro, h2SA, h3A, and C404Y) and helicity (A398P, L436P, G407A, and AAA), CSPs were monitored in 2D ^1^H,^15^N BEST-TROSY experiments of 25 μM and increasing the protein concentration up to 400 μM. The constructs used in this analysis were either ^15^N labeled or ^15^N,^13^C-double labeled. A threshold was defined as the mean of the lowest 25% of CSP values plus five times their SD; only residues with CSP ≥ threshold were used for the mean CSP. The error bars for the mean CSP depict the SEM, calculated as the SD of the retained residues divided by the square root of their count. To confirm that the changes in Tau-5* oligomerization caused by the L436P mutation result from disruptions in potential helical structure formation rather than altered hydrophobicity, we also investigated the effect of the T435P mutation on Tau-5* oligomerization (fig. S7E).

### Cysteine p*K*_a_ measurements

The p*K*_a_ values of individual Cys residues (C123, C404, and C518) were determined using ^15^N,^13^C-labeled single Cys fragments: Nt C123, Tau-5* C404, and Ct C518. The Cys ^13^C_β_ chemical shifts were measured in 70 μM Nt C123, 300 μM Tau-5* C404, and 150 μM Ct C518 samples. These measurements were conducted using ^1^H,^13^C-HSQC centered in the aliphatic region at varying pH from 6 to 12. All samples were prepared in the final buffer, containing 3 mM TCEP, to prevent disulfide bridge formation at high pH values.

The chemical shifts δ^13^C_β_ were fitted to the following equation, as previously reported (*72*)

$$\delta^{13}C_{\beta }=\frac{1}{1+10^{{pK}_{a}-\text{pH}}}+\delta_{\text{offset}}$$

The δ^13^C_β_ scale was normalized (0 < δ^13^C_β_ < 1) to correct for the slight differences in the chemical shift of the protonated state. The 95% confidence interval of the fitting was calculated by Monte Carlo error analysis.

### Dynamic light scattering

DLS measurements were performed using a Zetasizer Nano-S instrument (Malvern) equipped with a 633-nm He-Ne laser. All samples were freshly prepared before measurements, derived from stock solutions that had been filtered or centrifuged at 15,000 rpm for 10 min at 4°C (the supernatant was used after the concentration was determined) and equilibrated for 10 min. In addition, all samples of NR ADs and Tau-5* (except 1Tau-5* and 2Tau-5*) contained 0.5% DMSO-*d*_6_. Each sample was measured three times, and experiments were conducted at 5°C. The percentage population of each species was analyzed, as some samples exhibited a high polydispersity index. The hydrodynamic radius *R*_h_ of monomers was confirmed by comparing experimental values to the theoretically calculated *R*_h_, as reported previously (*73*).

### Microscale thermophoresis

Purified 2Tau-5* in a TCEP-free final buffer was labeled using the Monolith Protein Labeling Kit RED-NHS 2nd Generation (Nanotemper), following the manufacturer’s protocol. Measurements were performed on a Monolith NT.115 instrument, with data acquisition and analysis carried out using MO.Control and MO.Affinity Analysis softwares.

Labeled 2Tau-5* was used as the target at a concentration of 80 nM, with varying concentrations of EPI-001 (up to a solubility limit of 500 μM) at 25°C (fig. S7F). Samples contained 0.0125% Tween 20 and were loaded into Monolith Premium Capillaries (Nanotemper). The measurements were conducted at High MST power and 30% excitation power. Three measurements were performed for each sample, using independently prepared samples.

The binding curve was fitted to the analytical Hill equation to calculate the *K*_d_ (dissociation constant), assuming that the *F*_norm_ value at 500 μM represents the starting point of the bound plateau

$$c_{\text{drug}}=F_{\text{norm},\text{unbound}}+\frac{F_{\text{norm},\text{bound}}-F_{\text{norm},\text{unbound}}}{1+{\left(\frac{K_{d}}{c_{\text{drug}}}\right)}^{n}}$$

where *c*_drug_ represents the EPI-001 concentration, and *n* is the Hill coefficient.

### Mass spectrometry

A total of 25 μM protein samples were mixed with a 10-fold molar excess of the small molecule in the final buffer and adjusted to pH 8. The mixtures were incubated at 37°C from 1 to 24 hours. After the reaction, the pH of the samples was reduced to pH 7.4 using HCl. Subsequently, the samples were frozen and stored at −20°C before their measurement by intact MS.

In Fig. 4 (E and F), single Cys constructs were used—Tau-5* C404, Nt C123, Ct C518, and allTau-5 Cys mutants (C404, C448, and C518). For Fig. 4H, Tau-5* contained two Cys—C404 and C448—but no evidence of simultaneous covalent modification of both Cys was observed. Another experiment using Tau-5* with a sole Cys at C448 (Tau-5* C404Y and Tau-5* C404S mutants) showed no covalent modification, indicating no covalent reaction takes place when the Cys is at the C terminus.

Frozen samples were thawed and diluted to a final concentration of 5 μM using a 3% (v/v) acetonitrile (ACN) and 1% (v/v) formic acid (FA) aqueous solution. Samples were analyzed using an Acquity Ultra Performance chromatographic system coupled to an LCT Premier XE (time-of-flight) mass spectrometer (Waters Corp., Milford, MA, USA). A total of 40 pmol of the sample was injected using the Waters sample manager equipped with a Binary Solvent Manager. Protein content was separated on a BioSuite Phenyl 1000 column (Reverse phase chromatography, 2.0 mm by 75 mm, 10 μm, Waters Corp.) with a linear gradient of 5 to 80% B [A = 0.1% (v/v) FA in water, B = 0.1% (v/v) FA in ACN]. The column outlet interfaced directly with the electrospray ionization source of the spectrometer. The mass spectrometer operated in voltage analyzer mode with positive polarity. Capillary voltage and cone voltage were set at 3000 and 100 V, respectively. Desolvation temperature and source temperature were 300° and 120°C. Cone gas flow and desolvation gas flow were set at 50 and 600 liters/hour, respectively. Ion guide 1 and aperture 1 were set to 15 and 10 V, respectively. Full MS scans (400 to 4000 mass/charge ratio) were acquired using MassLynx software, V4.1.SCN704 (Waters Inc.). Manual deconvolution was performed using the MaxEnt 1 deconvolution algorithm, facilitated by V4.2.SCN982.

### Turbidity measurements

Turbidity measurements were performed to estimate *T*_c_ changes in allTau-5 induced by 250 μM EPI-001 or 5% allTau-5:EPI-001 adduct at a total protein concentration of 3 mg/ml and 100 mM NaCl. To measure protein constructs *T*_c_, concentrations of 2.4 mg/ml were used for AR AD fragments and Tau-5* mutants designed to modulate aromaticity and helicity while maintaining 3 mg/ml for NR ADs, unless otherwise specified. All samples were pretreated with 500 mM NaCl before experimentation. *T*_c_ values of AR AD* were used from the previous work (*15*). Notably, we did not observe GR AD condensate formation even when the NaCl concentration was increased up to 2 M. For Tau-5* mutants affecting aromatic content and patterning, we used 1.5 M NaCl, needed to induce condensate formation of the h2SA and h3A mutants. The AR fragment Ct contained a modified shorter polyG sequence comprising only four glycines (Ct 4G) to prevent protein aggregation (*63*).

The absorbances of the samples were recorded at 340 nm using 1-cm pathlength cuvettes and a Cary100 UV-visible spectrometer equipped with a multicell thermoelectric temperature controller. A gradual temperature increase at a ramp rate of 1°C/min was applied, and *T*_c_ values were determined by identifying the peaks of the first-order derivatives of the curves. SDs derived from three replicates were used to assess uncertainties.

### High-performance liquid chromatography

The concentrations of the allTau-5:EPI-001 adduct and noAro, along with the *c*_sat_ measurement for allTau-5 and the determination of the partition coefficient of EPI-001 into protein condensates, were assessed using the Agilent Technologies 1260 Infinity II HPLC with a Jupiter analytical C4 column from Phenomenex. H_2_O and ACN:H_2_O (9:1) were used as mobile phases, containing 0.1% (v/v) TFA.

### Saturation concentration measurement

Saturation concentration measurements were conducted to evaluate the alterations in the *c*_sat_ of allTau-5 induced by 250 μM EPI-001 or 100% allTau-5:EPI-001 adduct at a total protein concentration of 3 mg/ml. Formation of condensates was induced by 100 mM NaCl, and samples were incubated at 25°C for 5 min, followed by centrifugation for 2 min at 376*g*. The supernatant was injected into the HPLC system, and the corresponding peaks representing allTau-5 or allTau-5:EPI-001 adducts at 49.2% ACN:H_2_O (9:1) were integrated. Concentration was determined using calibration curves established for allTau-5 and allTau-5:EPI-001. The curves were generated by measuring the concentrations of individual samples using NanoDrop (Thermo Fisher Scientific) for allTau-5 and amino acid analysis for allTau-5:EPI-001. Subsequently, the samples and their dilutions were injected into the HPLC system to measure the peak integral dependency on concentrations.

### Measurement of small-molecule partition coefficients

To determine the partition coefficient of EPI-001 in protein condensates, samples were prepared at a protein concentration of 3 mg/ml. For Tau-5* mutants affecting helicity (A398P, L436P, WT, G407A, and AAA), condensation was induced with 500 mM NaCl in the presence of 250 μM EPI-001. For other constructs—including Tau-5* mutants altering aromaticity (h2SA, h3A, WT, and C404Y), AR AD fragments (AR AD, allTau-5, Tau-5*, and Ct 4G), and NR ADs (AR, MR, PR, and ERα)—condensation was induced with 1.75 M NaCl in the presence of 100 μM EPI-001. In addition, for the ADs of AR, MR, and ERα, the partition coefficient of EPI-001 was also assessed at 500 mM NaCl, using 250 μM EPI-001 (fig. S5E). Under these conditions, PR and GR did not form condensates.

EPI-001 was first added to the protein samples, followed by NaCl to induce condensation. This order of addition ensured that the compound was present during condensate nucleation and growth, thereby avoiding potential kinetic barriers to diffusion into preformed condensates and minimizing biases arising from condensate material properties. Samples were incubated for 5 min at 25°C and centrifuged at 376*g* for 2 min to separate the light and dense phases. The light phase was transferred to a different microcentrifuge tube, while the dense phase was resuspended in 25 μl of buffer containing 4 M urea to fully dissolve the condensates. From this suspension, 25 μl was transferred to a separate microcentrifuge tube for analysis, and the remaining dense-phase volume was measured. This residual volume was taken as the original dense-phase volume before dilution and was used to calculate the dilution factor. Equal volumes from the light and dense phases were subsequently analyzed by HPLC, with the EPI-001 signal monitored at 220 nm and eluting at 49.4% ACN:H_2_O (9:1). Integrating the corresponding peak of the ligand enabled the calculation of the ratio between the dense and light phases, determining the partition coefficient. Peak areas were used for quantitation in all cases; however, for NR ADs at 500 mM NaCl and for Tau-5* helicity mutants (500 mM NaCl), peak heights were used because certain samples showed peak-shape deviations that could bias area integration. For the dense phase, the measured signal was corrected by the dilution factor to account for resuspension in urea buffer. Notably, partition coefficient measurements were not conducted for the Nt fragment and GR AD due to the absence of observed condensate formation at concentrations up to 2 M NaCl.

### LacI tethering assay

The ADs of ERα, MR, and GR constructs were ordered from Twist Bioscience. The AD of AR and FUS LCD were amplified from plasmids used in our previous work (*15*), created in the D.H. laboratory (Addgene: 215637), respectively. The AD of PR was amplified from MCF7 cDNA. Primers used for the construct amplification are listed in the primer table in table S2. The amplified constructs were then inserted into the LacI-CFP-MCS plasmid pJM118 (*47*) using the NEBuilder HiFi DNA Assembly Master Mix kit [New England Biolabs (NEB), E2621X]. The insert:vector ratio used for assembly was 1:3 with 45 to 50 ng of vector. Cloned constructs were validated using Sanger sequencing.

Twenty-five thousand U2OS cells with a lacO array integrated into the genome (*44*) were seeded per well in Ibidi eight-well glass-bottom u-slides. The following day, cells were transfected with 100 ng of YFP-containing plasmid (YFP empty for control or YFP-RNAPII CTD) (*74*) and 75 ng of lacI-CFP (AD of NR or FUS LCD) plasmid using Lipofectamine 3000 (Thermo Fisher Scientific) according to the manufacturer’s protocol. Twenty-four hours after transfection, the medium was changed to medium containing either DMSO or 25 μM EPI-001. Cells were imaged approximately 24 hours after DMSO or EPI-001 treatment using a Zeiss LSM880 confocal microscope with a live cell imaging chamber set to 37°C and 5% CO_2_, using a Plan-Apochromat-63×/1.40 oil differential interference contrast (DIC) objective with a 2× zoom. Two biological replicates with 18 to 22 images were taken for each condition per replicate.

Acquired images were analyzed using FIJI (*75*). YFP and CFP intensities were measured in CFP tethers, which were manually selected for each cell. An area of the same size and shape was used for YFP background level normalization and enrichment calculation (intensity in tether/intensity in background) in each cell. The data presented in Fig. 5E was replotted for Fig. 5F, specifically for the DMSO with RNAPII CTD condition.

### Fluorescence microscopy

Purified proteins, including AR AD, allTau-5 C518, and Tau-5* C404, were labeled with DyLight 488 dye featuring a thiol-reactive maleimide (Thermo Fisher Scientific). The proteins and the dye were mixed at an approximate ratio of 1:10 in the final protein solution and left overnight at 4°C. Subsequently, isolation of the protein and labeled protein from the unbound dye was accomplished through SEC using HiLoad Superdex 75 or 200 pg 13/300 columns. Concentrations and conjugation efficiencies were assessed following the manufacturer’s guidelines. For fluorescence microscopy samples, a 1 μM dye concentration was used.

For fluorescence microscopy imaging, AR AD fragments (AR AD, allTau-5, and Tau-5*) were prepared at a concentration of 3 mg/ml, measured at 37°C, while Tau-5* mutants (C404Y, G407A, and WT) were at 2.4 mg/ml (200 μM), measured at 35°C. AR AD fragments were imaged both in the presence and absence of 250 μM EPI-001. Condensate formation was induced by adding 500 mM NaCl. NR ADs were assayed at 3 mg/ml with 0 or 500 mM NaCl. Because PR and GR ADs did not phase separate under these conditions, they were additionally tested at 1 M NaCl. Condensation assays for AR, MR, and ERα ADs were performed at 37°C, whereas PR and GR ADs were measured at 25°C.

In a chamber made of a slide and a coverslip with double-sided tape (3M 300 LSE high-temperature double-sided tape, 0.17-mm thickness), 1.8 μl of the sample was deposited. The coverslips were coated with polyethylene glycol–silane as per the protocol by Alberti *et al.* (*76*). Imaging was performed on the coverslip surface where the droplets were settled using a Zeiss LSM780 confocal microscope equipped with a Plan-ApoChromat 63x/1.4 oil objective lens. Laser power was adjusted to optimize the visualization of droplets.

Equivalent-sized droplets were selected for FRAP analysis, with the bleached area covering ~30% of their diameter. Intensity values were monitored across different regions of interest (ROIs): ROI1 corresponded to the bleached area, ROI2 encompassed the entire droplet, while ROI3 captured the background signal. The obtained data underwent processing via EasyFrap software, enabling the extraction of kinetic parameters such as the half-time of recovery (*t*_1/2_) (*77*).

### DIC microscopy

For DIC microscopy, AD domains of AR, PR, GR, and ERα were used at 16 μM, while the MR AD was used at 10 μM, all in the presence of 500 mM NaCl at 25°C. Samples were prepared in chambers composed of a slide, coverslip, and double-sided tape, as described above for the fluorescence microscopy. DIC images were acquired on an automated inverted Olympus IX81 microscope equipped with a 60×/1.42 oil Plan Apo N objective using Xcellence rt 1.2 software.

### Statistical analysis

Pairwise comparisons were conducted using a *t* test, except for the data in Fig. 5 (E and F) and extended data fig. S5 (G and H), for which Wilcoxon tests were used for statistical analysis. Significance levels were determined on the basis of the *P* values generated by the test, denoting significance as follows: ****P < 0.0001, ***P < 0.001, **P < 0.01, or *P < 0.05.

## Supplementary Materials

### This PDF file includes:

Figs. S1 to S7 Tables S1 and S2 References
