# Supplementary material for: Oligomerization enables the selective targeting of an intrinsically disordered region by a small molecule
Source: Sci Adv. 2026 Feb 27;12(9):eadz7400. doi: 10.1126/sciadv.adz7400 (PMC12947862; doi:10.1126/sciadv.adz7400)
Supplement: Supplementary file 1 — Figs. S1 to S7 Tables S1 and S2 References [file sciadv.adz7400_sm.pdf]

Science Advances
AAAS

Supplementary Materials for

Oligomerization enables the selective targeting of an intrinsically disordered region by a small molecule

Stasė Bielskutė-García *et al.*

Corresponding author: Xavier Salvatella, xavier.salvatella@irbbarcelona.org

Sci. Adv. **12**, eadz7400 (2026)
DOI: 10.1126/sciadv.adz7400

This PDF file includes:

Figs. S1 to S7
Tables S1 and S2
References

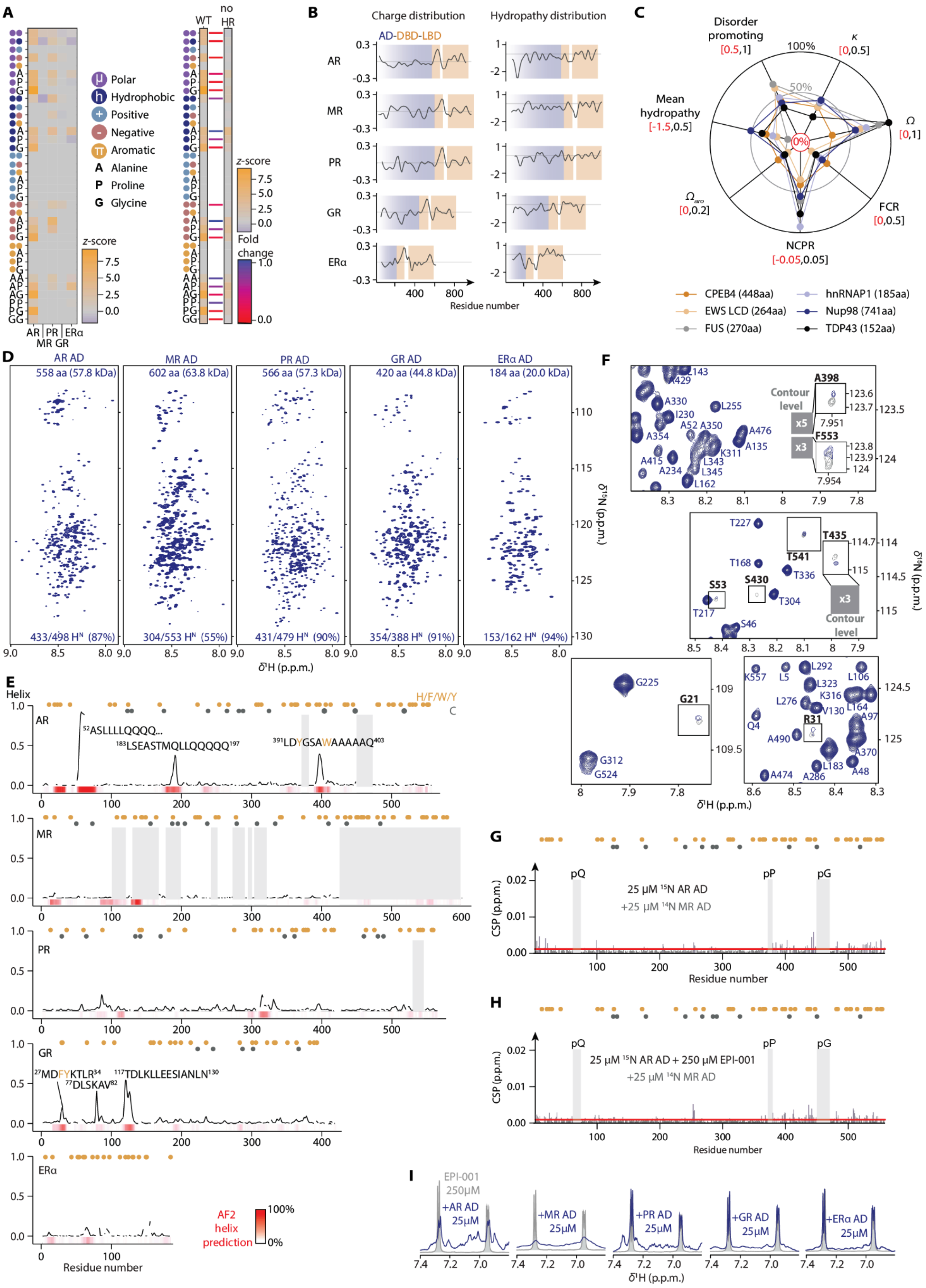
A
Polar
Hydrophobic
Positive
Negative
Aromatic
Alanine
Proline
Glycine
z-score
AR PR ERα
MR GR
WT
no HR
Fold change
B
Charge distribution
Hydropathy distribution
AD-DBD-LBD
AR
MR
PR
GR
ERα
Residue number
C
Disorder promoting [0.5,1]
κ [0,0.5]
Ω [0,1]
FCR [0,0.5]
NCPR [-0.05,0.05]
Ωaro [0,0.2]
Mean hydropathy [-1.5,0.5]
100%
50%
0%
CPEB4 (448aa)
EWS LCD (264aa)
FUS (270aa)
hnRNAP1 (185aa)
Nup98 (741aa)
TDP43 (152aa)
D
AR AD 558 aa (57.8 kDa)
MR AD 602 aa (63.8 kDa)
PR AD 566 aa (57.3 kDa)
GR AD 420 aa (44.8 kDa)
ERα AD 184 aa (20.0 kDa)
433/498 Hᴺ (87%)
304/553 Hᴺ (55%)
431/479 Hᴺ (90%)
354/388 Hᴺ (91%)
153/162 Hᴺ (94%)
δ¹H (p.p.m.)
δ¹⁵N (p.p.m.)
E
Helix
H/F/W/Y
C
⁵²ASLLLLQQQQ...
¹⁸³LSEASTMQLLQQQQQ¹⁹⁷
³⁹¹LDYGSAWAAAAAQ⁴⁰³
²⁷MDFYKTLR³⁴
⁷⁷DLSKAV⁸²
¹¹⁷TDLKLLEESIANLN¹³⁰
AF2 helix prediction
Residue number
F
Contour level
x5
x3
A398
F553
T435
T541
S53
S430
G21
R31
δ¹H (p.p.m.)
δ¹⁵N (p.p.m.)
G
pQ
pP
pG
25 μM ¹⁵N AR AD
+25 μM ¹⁴N MR AD
CSP (p.p.m.)
Residue number
H
25 μM ¹⁵N AR AD + 250 μM EPI-001
+25 μM ¹⁴N MR AD
I
EPI-001 250μM
+AR AD 25μM
+MR AD 25μM
+PR AD 25μM
+GR AD 25μM
+ERα AD 25μM
δ¹H (p.p.m.)

Fig. S1.

(**A**) (*Left*) Clustering analysis of non-random amino acid sequences across the activation domains of the nuclear receptor family (*61*). Positive *z*-scores (orange) indicate a linear clustering ("blocky"), and negative values (blue) indicate uniform dispersion ("well-mixed"). These parameters were calculated for residue classes that are present in a proportion that is greater than 10%. (*Right*) NARDINI analysis (*61*) of the sequence segregation of certain amino acid-type classes in AR AD in the presence and absence of homorepeats (poly-glutamine, -proline and -glycine). Positive *z*-scores (orange) indicate a blockiness of these amino acids compared to a random distribution of *in silico* generated 10^6 sequences that retain the overall composition. In general, the sequence segregation is reduced by eliminating the homorepeats. (**B**) Sequence profile of the physicochemical properties (charge and hydropathy). (**C**) Overall sequence properties for the IDRs of TDP-43, FUS, EWS low-complexity domain, Nup98, hnRNAP1, and CPEB4. Mean hydropathy; Fraction of disorder promoting residues in the sequence; κ and Ω values describe the segregation of charged and proline residues (*58*, *78*); FCR, fraction of charged residue; NCPR, net charge per residue; Aromatic clustering measures the normalized patterning of aromatic residues (*60*). (**D**) 2D ^{1}H-^{15}N NMR correlation spectra of the different NR ADs. The total amount of amide protons assigned, either from sequential assignment or indirectly from a divide-and-conquer approach or urea titration, is indicated. For MR AD, the spectrum shown is in the absence of urea; assignment coverage increases to 496/553 (90%) in the presence of 2 M urea. The narrow dispersion in the ^{1}H dimension is an indicative NMR signature of IDRs. The heterogeneous intensities are a typical property of IDRs mediating intermolecular homotypic interactions with other protein molecules. (**E**) NMR-derived helical content of NR ADs, using the δ2D algorithm (*71*). Helical populations for AR AD were extracted from the chemical shift of separate constructs (*15*). AlphaFold predicted helical populations are indicated as a red barcode in the bottom of each plot. (**F**) Representative NMR signals of the AR AD (grey) that experience significant CSP in the presence of 10 molar equivalents of EPI-001 (blue). (**G**) CSPs extracted from 2D ^{1}H-^{15}N NMR correlation spectra of AR AD induced by 1 molar equivalent of MR AD. (**H**) CSPs induced by MR AD in the 2D ^{1}H-^{15}N NMR correlation spectra of 1 molar equivalent of AR AD and 10 equimolar EPI-001. (**I**) Aromatic regions of 1D ^{1}H spectra of EPI-001 in the absence (grey) or presence (blue) of a 0.1 molar equivalent of the corresponding AD. (**G, H**) The red line represents the significant threshold calculated as the mean plus five standard deviations of the first quartile of CSPs. (**E, G, H**) Orange and grey dots above the residue plots indicate the position of aromatic or cysteine residues, respectively. Grey shaded boxes indicate residues unassignable in the apo spectra due to intrinsic broadening or overlap and therefore not evaluated for CSPs.

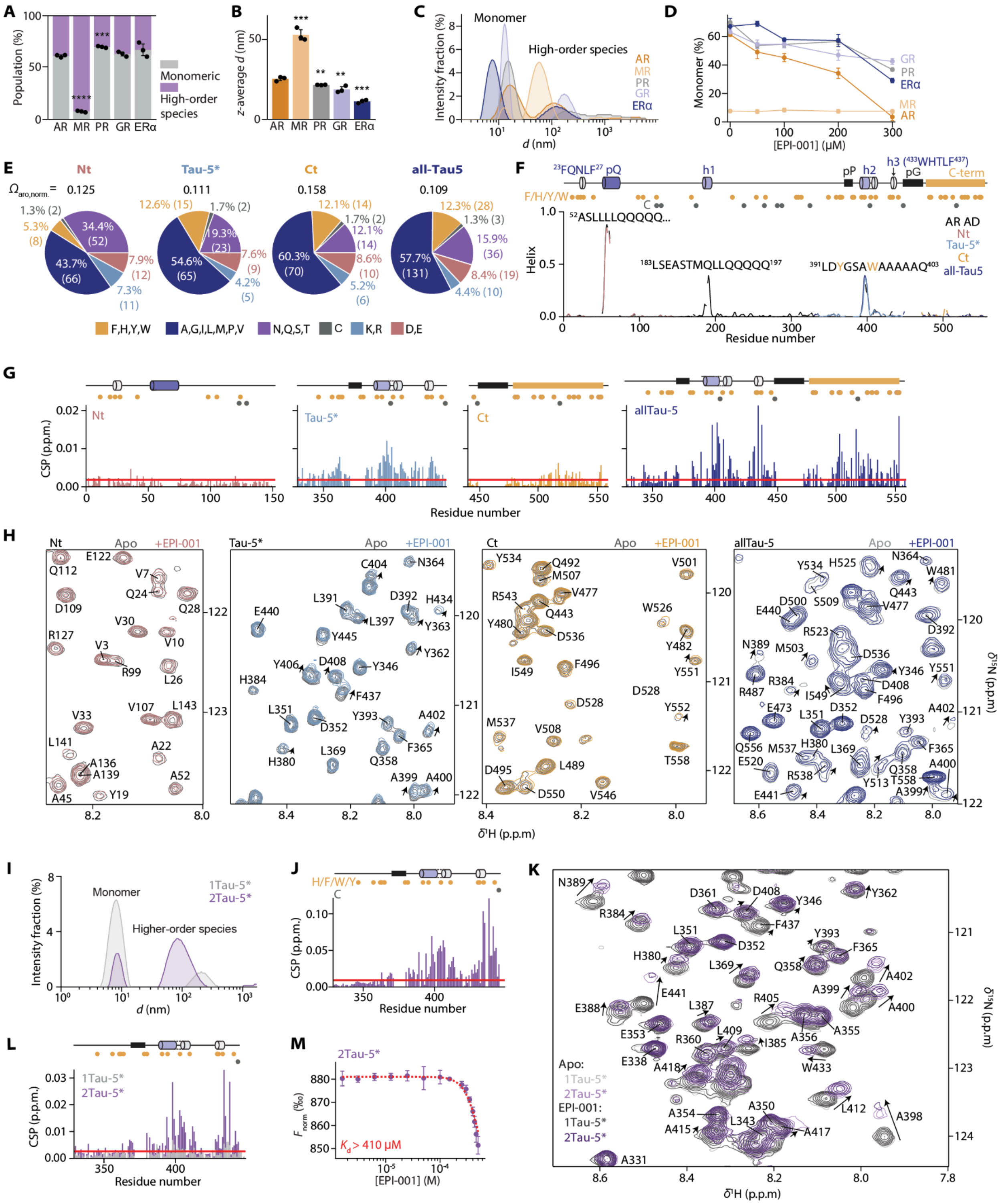

Fig. S2.
(**A**) Percentage of monomeric and higher-order species and (**B**) *Z*-average hydrodynamic diameter in NR AD samples analyzed by DLS at 2 mg/mL. (**C**) Corresponding DLS intensity measurement of NR ADs. (**D**) Percentage of monomer in 2 mg/mL NR AD samples at varying concentrations of EPI-001. The remaining percentage represents high-order species measured by DLS. (**E**) Amino acid composition of AR AD fragments depicted for six different residue types:

polar (purple), cysteine (grey), positive charge (light blue), negative charge (pink), aromatic (orange), and hydrophobic (blue). (**F**) Annotation of short helical motifs in the AR AD. The plots display the helical propensity of the AR AD and its fragments, derived from NMR backbone chemical shifts; propensities were estimated with the δ2D algorithm (*71*). The ^{23}FQNLF^{27} and ^{433}WHTLF^{437}(h3) motifs are known to fold upon binding to cellular partners (*32*). Orange and grey circles indicate the positions of aromatic and cysteine residues, respectively. (**G**) CSPs induced by 250 μM EPI-001 were measured in 2D ^{1}H-^{15}N NMR correlation spectra of AR AD fragments at a protein concentration of 25 μM. (**H**) NMR spectral region of the AR AD fragments in the presence (colour) and absence (gray) of the compound. (**I**) DLS intensity measurement of 1Tau-5* and 2Tau-5* at a concentration of 2.44 mg/mL. (**J**) CSPs obtained by comparing the 2D ^{1}H-^{15}N NMR correlation spectra of 1Tau-5* and 2Tau-5* at 0.31 mg/mL. (**K**) Representative NMR signals of 1Tau-5* and 2Tau-5* in the absence and presence of EPI-001 measured in 2D ^{1}H-^{15}N NMR correlation spectra. (**L**) Per-residue CSPs in 2D ^{1}H-^{15}N NMR correlation spectra of 1Tau-5* and 2Tau-5* at 0.31 mg/mL after adding 250 μM EPI-001. In both cases, the ratio corresponds to 1:10 EPI-001 interaction sites on the protein to EPI-001. (**M**) MST binding curves of EPI-001 with 2Tau-5* at a fixed protein concentration of 80 nM. The curve was fitted using the analytical Hill equation, and the K_d value was calculated under the assumption that a plateau is reached at the highest measured concentration of EPI-001. (**A, B, D, M**) Error bars indicate the standard deviations (n = 3). (**G, J, L**) The red line represents the significant threshold calculated as the mean plus five standard deviations of the first quartile of CSPs.

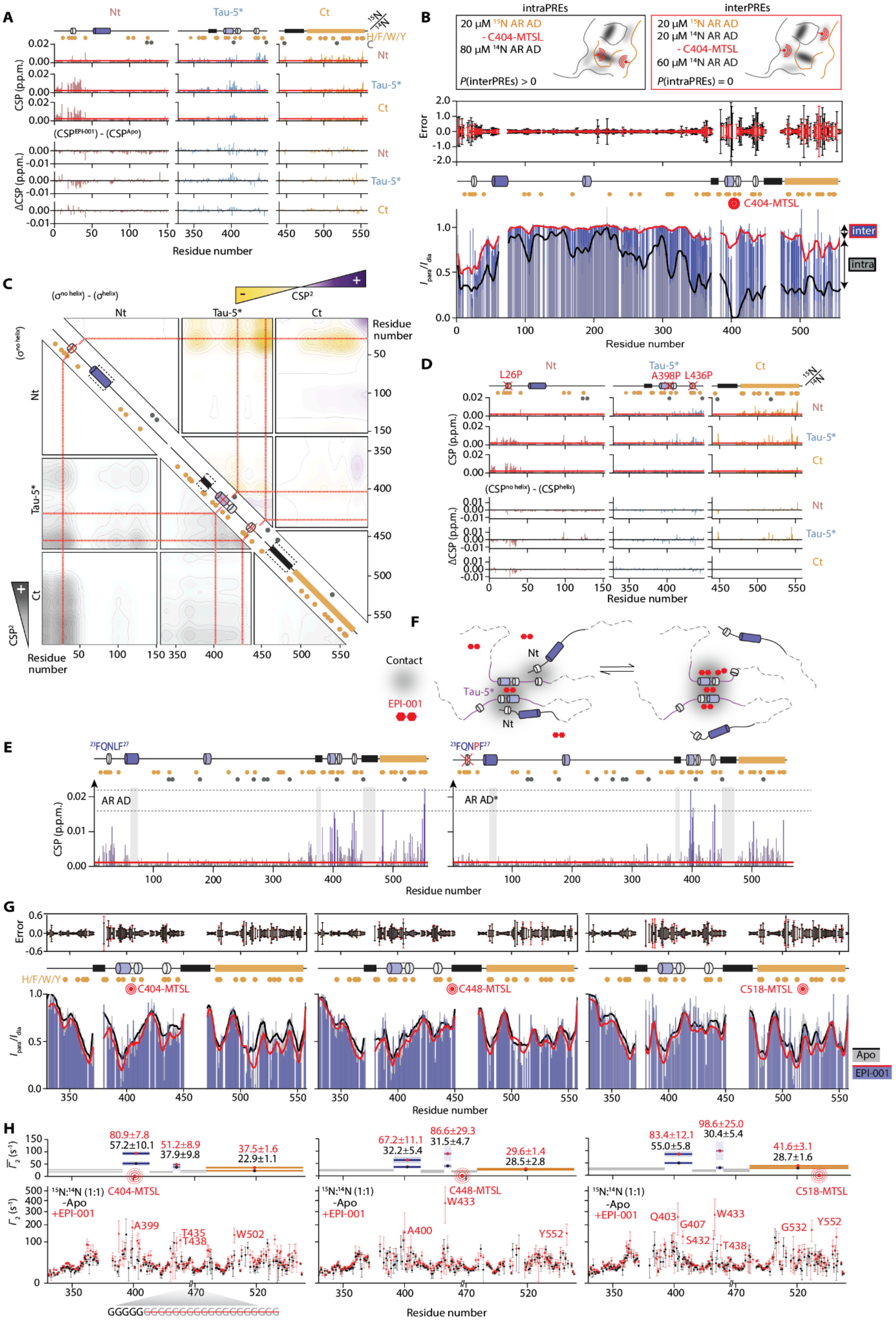
A
Nt
Tau-5*
Ct
15N/14N
H/F/W/Y
C
CSP (p.p.m.)
(CSP^EPI-001) - (CSP^Apo)
ΔCSP (p.p.m.)
Residue number
B
intraPREs
20 μM 15N AR AD - C404-MTSL
80 μM 14N AR AD
P(interPREs) > 0
interPREs
20 μM 15N AR AD
20 μM 14N AR AD - C404-MTSL
60 μM 14N AR AD
P(intraPREs) = 0
Error
C404-MTSL
I_para/I_dia
inter
intra
Residue number
C
(σno helix) - (σhelix)
CSP²
Residue number
D
L26P
A398P
L436P
CSP (p.p.m.)
(CSPno helix) - (CSPhelix)
ΔCSP (p.p.m.)
Residue number
E
23FQNLF27
23FQNPF27
AR AD
AR AD*
CSP (p.p.m.)
Residue number
F
Contact
EPI-001
Nt
Tau-5*
G
Error
H/F/W/Y
C404-MTSL
C448-MTSL
C518-MTSL
I_para/I_dia
Apo
EPI-001
Residue number
H
Γ̄2 (s⁻¹)
80.9±7.8
57.2±10.1
51.2±8.9
37.9±9.8
37.5±1.6
22.9±1.1
67.2±11.1
32.2±5.4
86.6±29.3
31.5±4.7
29.6±1.4
28.5±2.8
83.4±12.1
55.0±5.8
98.6±25.0
30.4±5.4
41.6±3.1
28.7±1.6
Γ2 (s⁻¹)
15N:14N (1:1)
-Apo
+EPI-001
A399
T435
T438
W502
C448-MTSL
W433
A400
Y552
Q403
G407
S432
W433
T438
G532
Y552
GGGGGGGGGGGGGGGGGGGGGGG
Residue number

Fig. S3.

(**A**) (*Top*) CSPs residue plots obtained from 2D ^{1}H-^{15}N NMR correlation spectra, used to calculate the CSP matrix in the absence of EPI-001 (Fig. 3B) and (*bottom*) difference CSP residue plots used to calculate the ΔCSP matrix values (Fig. 3C). Orange and grey circles indicate the positions of aromatic and cysteine residues, respectively. (**B**) Intra and intermolecular interactions within AR AD analyzed using PRE NMR experiments. *(Top)* Schematic of the experimental design. "*P*" represents the probability of detecting inter-PREs arising from interactions between two ^{15}N-labelled AR AD molecules in an experiment designed to measure intramolecular interactions, and intra-PREs in an experiment designed to detect intermolecular interactions. *(Middle)* Error bars are inversely proportional to the propagated signal-to-noise ratio of individual resonances. *(Bottom)* PRE intensity ratio graphs (I_{para}/I_{dia}) report spatial proximity within AR AD, with a black line (with gray bars) showing intramolecular PREs and red (with blue bars) showing intermolecular PREs. Lower I_{para}/I_{dia} values correspond to stronger PRE effects, indicating a closer proximity to the spin label introduced at C404 (all other cysteines were replaced by serines). (**C**) CSP matrix between equimolar mixtures of AR AD fragments where the helical elements were mutated (L26P in Nt, A398P and L436P in Tau-5*). CSP^2 in the absence of helical content is shown in grey. For visualization, the same color scale as Fig. 3B was used to enable direct comparison. Differences in the CSP^2 upon transient helix formation are shown in the upper matrix. Dashed black outlines on the fragments schemes mark regions which were not assigned. (**D**) CSPs residue plots, obtained from 2D ^{1}H-^{15}N NMR correlation spectra, used to generate the CSP matrices shown in Fig. S3C. (**E**) Per-residue CSPs induced by 10 molar equivalents of EPI-001 in 2D ^{1}H-^{15}N NMR correlation spectra of AR AD and AR AD* (*15*). AR AD* contains the L26P mutation, which prevents secondary structure formation within the ^{23}FQNLF^{27} motif. (**F**) Scheme illustrating EPI-001 competition with the ^{23}FQNLF^{27} motif for binding to its target site. (**G, H**) Intermolecular interactions between allTau-5 molecules monitored via PRE NMR experiments in the absence (black) and presence of 1 molar equivalent of the ligand (red). (**G**) *(Top)* Error bars are inversely proportional to the propagated signal-to-noise ratio of individual resonances. *(Bottom)* PRE intensity ratio graphs (I_{para}/I_{dia}) illustrate intermolecular interactions in allTau-5 with a paramagnetic spin label at different cysteine positions, while remaining cysteines were mutated to serine. (**H**) Error bars indicate the standard error from the exponential fit. The average values of Γ_2^{HN} for certain regions are shown in upper plots. Error bars represent the standard deviations. (**A, D, E**) The red line represents the significant threshold calculated as the mean plus five standard deviations of the first quartile of CSPs.

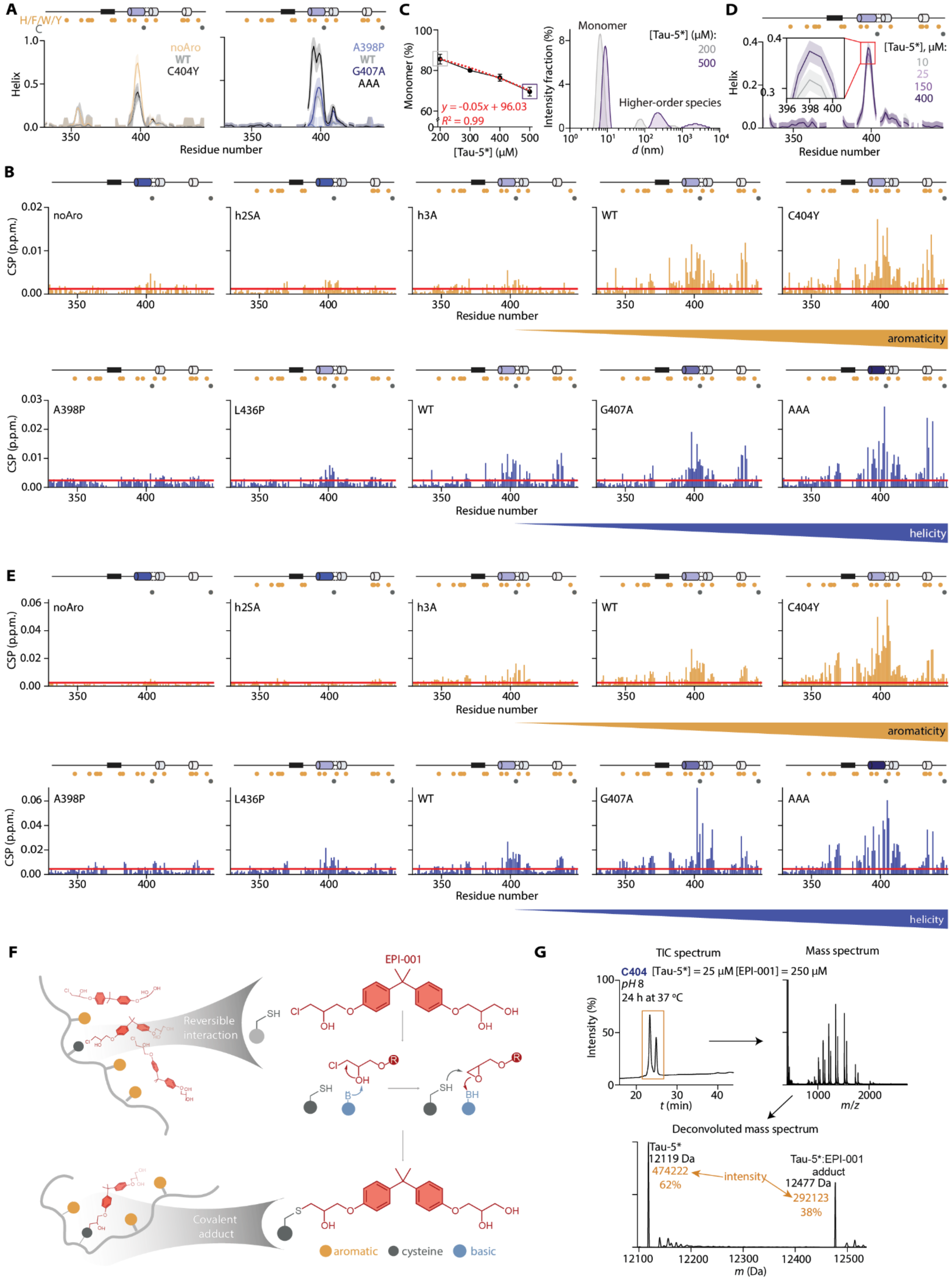
A
H/F/W/Y
C
Helix
noAro
WT
C404Y
A398P
WT
G407A
AAA
Residue number
C
Monomer (%)
y = -0.05x + 96.03
R² = 0.99
[Tau-5*] (μM)
Intensity fraction (%)
Monomer
[Tau-5*] (μM):
200
500
Higher-order species
d (nm)
D
[Tau-5*], μM:
10
25
150
400
B
CSP (p.p.m.)
noAro
h2SA
h3A
WT
C404Y
aromaticity
A398P
L436P
G407A
AAA
helicity
E
F
EPI-001
Reversible interaction
Covalent adduct
aromatic
cysteine
basic
G
TIC spectrum
Mass spectrum
C404 [Tau-5*] = 25 μM [EPI-001] = 250 μM
pH 8
24 h at 37 °C
Intensity (%)
t (min)
m/z
Deconvoluted mass spectrum
Tau-5*
12119 Da
474222
62%
intensity
Tau-5*:EPI-001 adduct
12477 Da
292123
38%
m (Da)

Fig. S4.

(**A**) NMR-derived helical content of Tau-5* mutants, computed from backbone chemical shifts using the *δ*2D algorithm (*71*). Orange and grey dots indicate the positions of aromatic and cysteine residues, respectively. (**B**) CSPs detected in the 2D ^{1}H-^{15}N NMR correlation spectra of Tau-5* mutants, engineered to modulate aromaticity or helicity, in response to 1 molar equivalent of EPI-001. (**C**) (*Left*) Percentage of monomer at different concentrations of Tau-5* samples measured by DLS, with the remaining percentage corresponding to higher-order species. Error bars indicate the standard deviations (n = 3). (*Right*) DLS intensity fraction measurement of Tau-5* at concentrations of 200 µM and 500 µM. (**D**) NMR-derived helical content of Tau-5* at various concentrations, computed from backbone chemical shifts using the *δ*2D algorithm (*71*). The enlarged region shows the helical content of Tau-5* at 10 and 400 µM. (**E**) CSPs detected in 2D ^{1}H-^{15}N NMR correlation spectra of Tau-5* mutants induced by increasing concentrations of the protein from 25 to 400 µM. These CSPs indicate regions involved in protein oligomerization. (**F**) Scheme showing the covalent reaction mechanism between the chlorohydrin warhead and the thiol group of cysteines. In a first step, the proximity between both chemical groups is induced by the transient binding of the aromatic side-chains. (**G**) Schematic workflow of the quantification of covalent adduct formation by HPLC-MS. The adduct and the free protein were separated by liquid chromatography and the mass of each peak was determined by intact MS. The peak intensity (integral) of the deconvoluted spectra was used to calculate the relative populations of protein and adduct. (**B, E**) The red line represents the significant threshold calculated as the mean plus five standard deviations of the first quartile of CSPs.

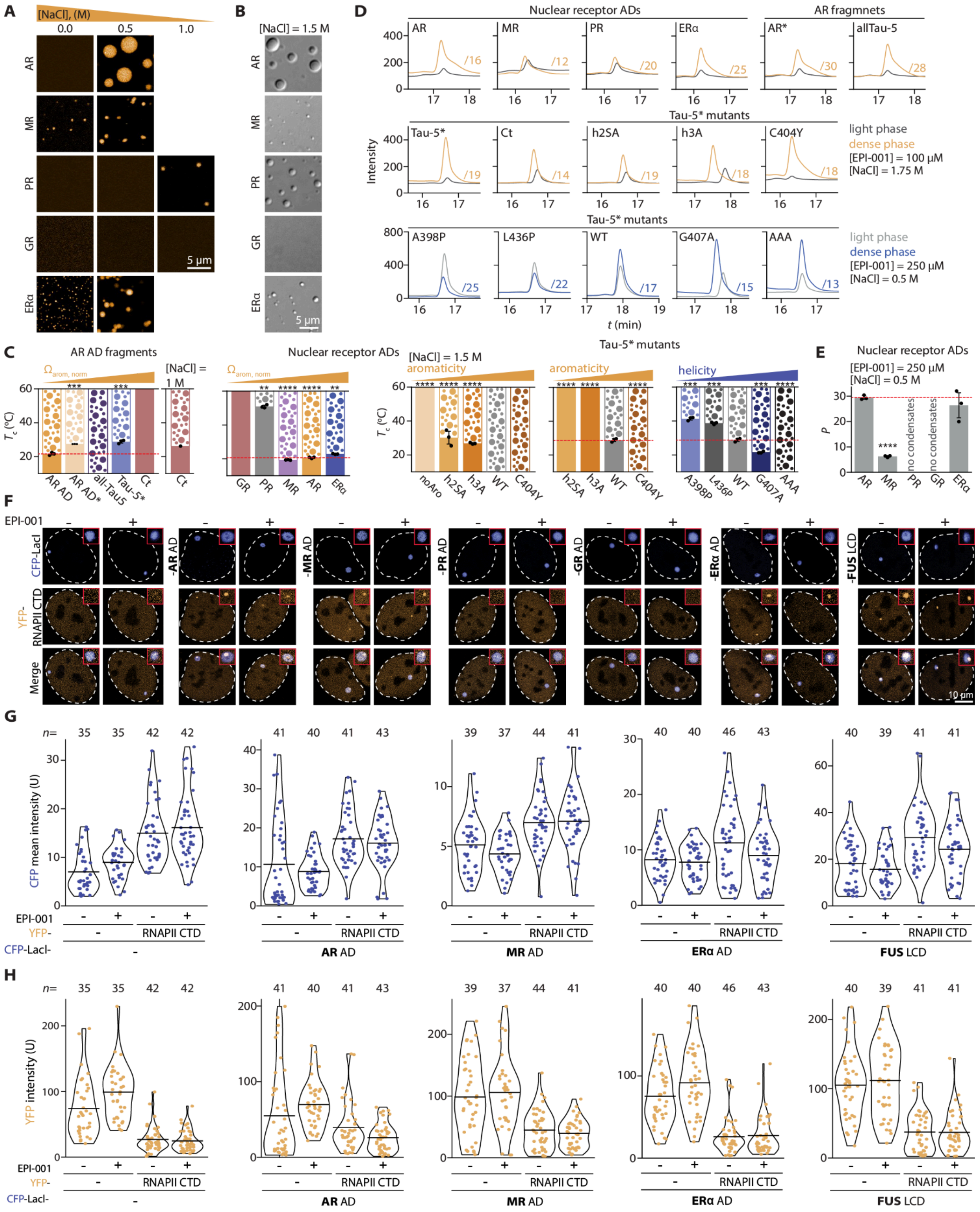

Fig. S5.

(**A**) Fluorescence microscopy images of NR AD constructs at different NaCl concentrations, showing condensate formation *in vitro*. ADs of AR, MR, and ERα were imaged at 37 °C, whereas PR and GR were imaged at 25 °C. (**B**) DIC microscopy images of NR AD constructs.

All constructs were used at 16 μM, except MR, which was used at 10 μM. Proteins were imaged at 25 °C. (**C**) T_c of AR AD fragments, NR ADs and Tau-5* mutants at 500 mM NaCl, unless otherwise specified. Errors shown on dots are standard deviations (*n* = 3). (**D**) HPLC chromatograms showing the EPI-001 signal in the dense and light phases of the proteins. The numbers indicate the dilution factor of the dense phase using a buffer containing 4 M urea for dissolution. (**E**) EPI-001 partition coefficients into condensates of NR ADs. Errors shown on dots are standard deviations (*n* = 3). (**F**) Representative images of cells co-transfected with YFP-RNAPII CTD and CFP-LacI-(NR AD or FUS LCD). Cells were treated either in the absence (-) or presence (+) of 25 μM EPI-001. Each image depicts one example nucleus, with the nuclear contour highlighted by a white dashed line. Within the red squares, a zoomed-in version of the CFP focus is displayed. Notably, the RNAPII CTD signal does not entirely overlap with the tether, consistent with known characteristics of RNAPII CTD recruitment in this assay (*45*, *46*). The two sets of far left panels are reproductions of the panels shown in Fig. 5D in the main text. (**G**) Comparative analysis of CFP signal intensity within the tethers of the NR ADs and FUS LCD foci. (**H**) Comparison of YFP (-) and RNAPII CTD-YFP signal intensities in the cellular background of cells transfected with different NR ADs and FUS LCD tethers. (**G, H**) Each data point represents a single tether/cell.

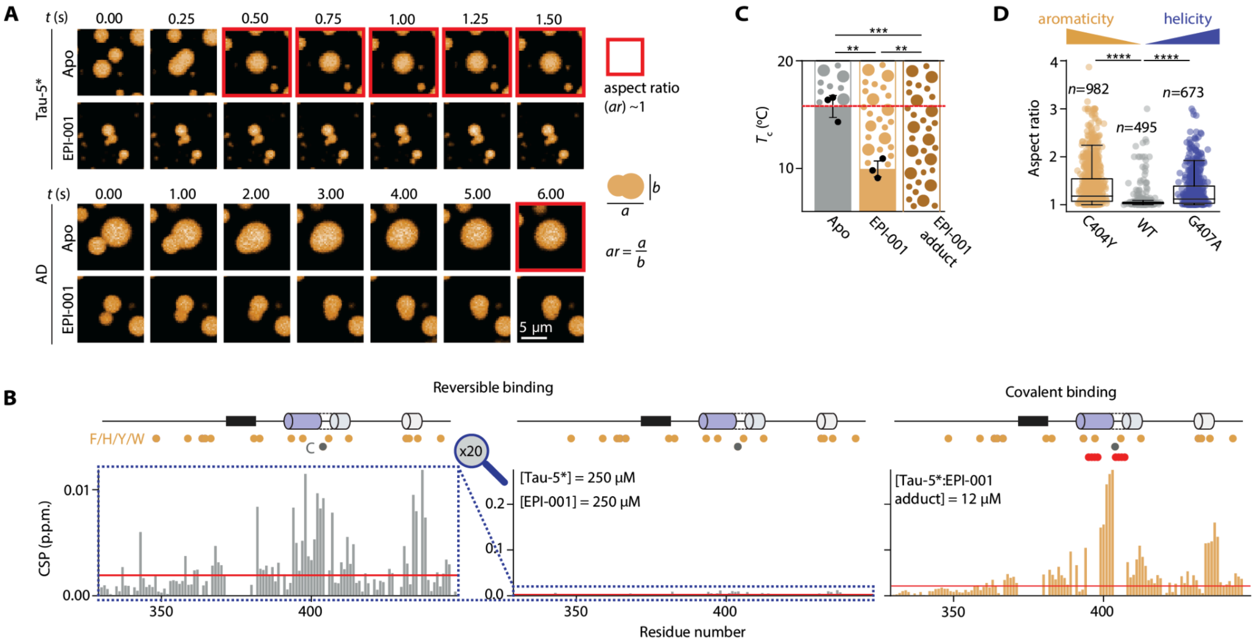

Fig. S6.
(**A**) Fusion events monitored at different time points of AR AD and Tau-5* condensates in the presence and absence of EPI-001 *in vitro*. Spherical fused droplets are highlighted in red. (**B**) CSPs in 2D ^{1}H-^{15}N NMR correlation spectra of Tau-5* induced by the covalent and reversible interaction of 1 molar equivalent EPI-001, respectively. Covalent binding induced CSPs approximately 20 times higher than reversible interaction. Orange and grey circles indicate the positions of aromatic and cysteine residues, respectively. Red circles indicate not assigned residues due to line broadening. The red line represents the significant threshold calculated as the mean plus five standard deviations of the first quartile of CSPs. (**C**) T_c measurements of allTau-5 in the presence and absence of EPI-001 (ratio 1:1), and allTau-5:EPI-001 adduct. Reduced T_c indicates an enhancement of condensation by the ligand. Errors are standard deviations (n = 3). (**D**) Aspect ratio quantification of Tau-5* mutants based on fluorescence microscopy images shown in Fig. 6G.

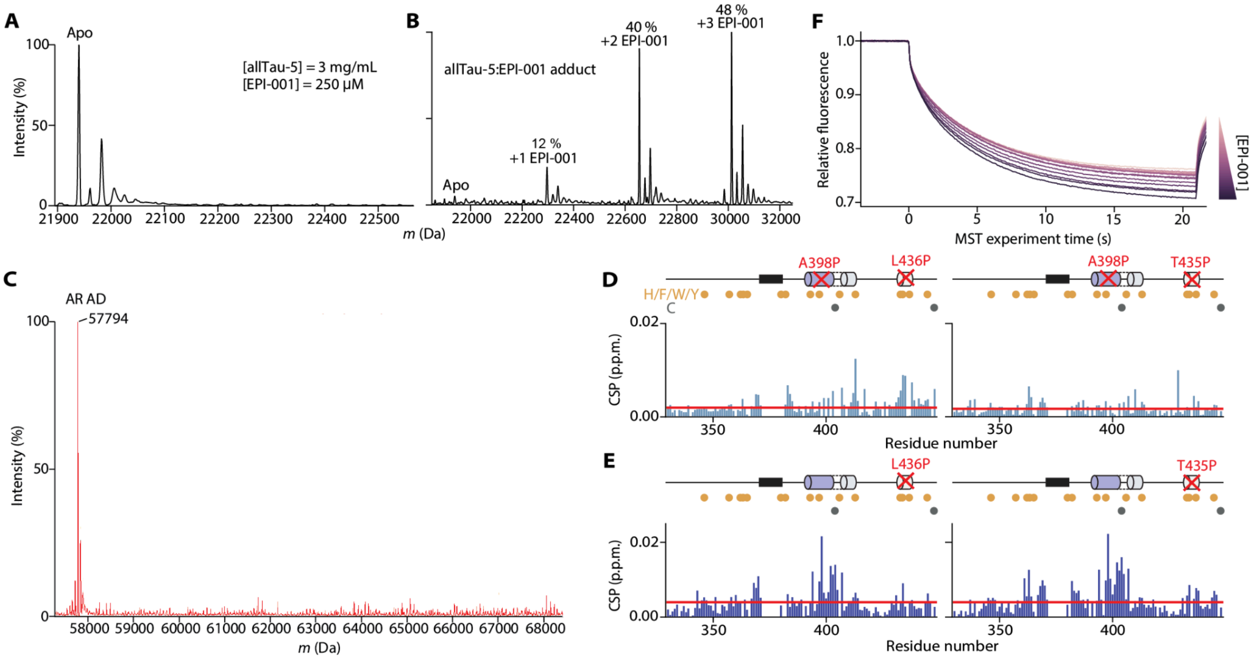

Fig. S7.
(**A**) Deconvoluted mass spectra of allTau-5 incubated with EPI-001 for 30 minutes at conditions that were used to study the condensates by fluorescence microscopy. No protein adduct was observed, indicating that the observed effects report exclusively on the reversible binding. **(B)** Deconvoluted mass spectra of allTau-5:EPI-001 adduct, revealing covalent interaction of EPI-001 with all three cysteines in allTau-5. (**C**) Deconvoluted mass spectra of AR AD incubated with EPI-001 for 48 hours under conditions consistent with those used for NMR studies of EPI-001 binding: pH 7.4, 5 °C. No peaks corresponding to a +358.43 Da mass shift were detected. AR AD contains 11 cysteines. (**D**) CSPs detected in 2D ^{1}H-^{15}N NMR correlation spectra of Tau-5* mutants A398P+L436P and A398P+T435P by increasing the protein concentration from 200 µM to 400 µM. Orange and grey dots indicate the positions of aromatic and cysteine residues, respectively. (**E**) CSPs observed in the NMR spectra of Tau-5* mutants L436P and T435P by increasing the protein concentration from 25 µM to 400 µM. (**F**) MST traces of 80 nM 2Tau-5* in the presence of increased concentrations of EPI-001 (from 1.6 to 500 µM). (**D, E**) The red line represents the significant threshold calculated as the mean plus five standard deviations of the first quartile of CSPs.

Table S1.

The sequences of proteins used for the *in vitro* experiments. Mutation positions are in bold font and underlined.

Name	Protein sequence
AR AD	[1]MEVQLGLGRVYPRPPSKTYRGAFQNLFQSVREVIQNPGPRHPEAASAAPPGASLLLL<br>QQQQQQQQQQQQQQQQQQQQQETSPRQQQQQQGEDGSPQAHRRGPTGYLVLDEEQ<br>QPSQPQSALECHPERGCVPEPGAAVAASKGLPQQLPAPPDEDDSAAPSTLSLLGPTFPG<br>LSSCSADLKDILSEASTMQLLQQQQQEAVSEGSSSGRAREASGAPTSSKDNYLGGTSTI<br>SDNAKELCKAVSVSMGLGVEALEHLSPGEQLRGDCMYAPLLGVPPAVRPTPCAPLAE<br>CKGSLLDDSAGKSTEDTAEYSPFKGGYTKGLEGESLGCSGSAAAGSSGTLELPSTLSL<br>YKSGALDEAAAYQSRDYYNFPLALAGPPPPPPPPHPHARIKLENPLDYGSAWAAAAA<br>QCRYGDLASLHGAGAAGPGSGSPSAAASSSWHTLFTAEEGQLYGPCGGGGGGGGGG<br>GGGGGGGGGGGGGGEAGAVAPYGYTRPPQGLAGQESDFTAPDVWYPGGMVSRVPY<br>PSPTCVKSEMGPWMDSYSGPYGDMRLETARDHVLPIDYYFPPQKT[558]
AR AD* (L26P)	[1]MEVQLGLGRVYPRPPSKTYRGAFQN**P**FQSVREVIQNPGPRHPEAASAAPPGASLLLL<br>QQQQQQQQQQQQQQQQQQQQQETSPRQQQQQQGEDGSPQAHRRGPTGYLVLDEEQ<br>QPSQPQSALECHPERGCVPEPGAAVAASKGLPQQLPAPPDEDDSAAPSTLSLLGPTFPG<br>LSSCSADLKDILSEASTMQLLQQQQQEAVSEGSSSGRAREASGAPTSSKDNYLGGTSTI<br>SDNAKELCKAVSVSMGLGVEALEHLSPGEQLRGDCMYAPLLGVPPAVRPTPCAPLAE<br>CKGSLLDDSAGKSTEDTAEYSPFKGGYTKGLEGESLGCSGSAAAGSSGTLELPSTLSL<br>YKSGALDEAAAYQSRDYYNFPLALAGPPPPPPPPHPHARIKLENPLDYGSAWAAAAA<br>QCRYGDLASLHGAGAAGPGSGSPSAAASSSWHTLFTAEEGQLYGPCGGGGGGGGGG<br>GGGGGGGGGGGGGGEAGAVAPYGYTRPPQGLAGQESDFTAPDVWYPGGMVSRVPY<br>PSPTCVKSEMGPWMDSYSGPYGDMRLETARDHVLPIDYYFPPQKT[558]
AR C404	[1]MEVQLGLGRVYPRPPSKTYRGAFQNLFQSVREVIQNPGPRHPEAASAAPPGASLLLL<br>QQQQQQQQQQQQQQQQQQQQQETSPRQQQQQQGEDGSPQAHRRGPTGYLVLDEEQ<br>QPSQPQSALE*HPERG*VPEPGAAVAASKGLPQQLPAPPDEDDSAAPSTLSLLGPTFPGL<br>SS*SADLKDILSEASTMQLLQQQQQEAVSEGSSSGRAREASGAPTSSKDNYLGGTSTIS<br>DNAKEL*KAVSVSMGLGVEALEHLSPGEQLRGD*MYAPLLGVPPAVRPTP*APLAE*K<br>GSLLDDSAGKSTEDTAEYSPFKGGYTKGLEGESLG*SGSAAAGSSGTLELPSTLSLYKS<br>GALDEAAAYQSRDYYNFPLALAGPPPPPPPPHPHARIKLENPLDYGSAWAAAAAQCR<br>YGDLASLHGAGAAGPGSGSPSAAASSSWHTLFTAEEGQLYGP*GGGGGGGGGGGG<br>GGGGGGGGGGGEAGAVAPYGYTRPPQGLAGQESDFTAPDVWYPGGMVSRVPYPSPT<br>*VKSEMGPWMDSYSGPYGDMRLETARDHVLPIDYYFPPQKT[558]
MR AD	[1]METKGYHSLPEGLDMERRWGQVSQAVERSSLGPTERTDENNYMEIVNVSCVSGAIPN<br>NSTQGSSKEKQELLPCLQQDNNRPGILTSDIKTELESKELSATVAESMGLYMDSVRDA<br>DYSYEQQNQQGSMSPAKIYQNVEQLVKFYKGNGHRPSTLSCVNTPLRSFMSDSGSSV<br>NGGVMRAVVKSPIMCHEKSPSVCSPLNMTSSVCSPAGINSVSSTTASFGSFPVHSPITQ<br>GTPLTCSPNVENRGSRSHSPAHASNVGSPLSSPLSSMKSSISSPPSHCSVKSPVSSPNNV<br>TLRSSVSSPANINNSRCSVSSPSNTNNRSTLSSPAASTVGSICSPVNNAFSYTASGTSAGS<br>STLRDVVPSPDTQEKGAQEVPFPKTEEVESAISNGVTGQLNIVQYIKPEPDGAFSSSCL<br>GGNSKINSDSSFSVPIKQESTKHSCSGTSFKGNPTVNPFPFMDGSYFSFMDDKDYYSLS<br>GILGPPVPGFDGNCEGSGFPVGIKQEPDDGSYYPEASIPSSAIVGVNSGGQSFHYRIGA<br>QGTISLSRSARDQSFQHLSSFPPVNTLVESWKSHGDLSSRRSDGYPVLEYIPENVSSSTL<br>RSVSTGSSRPSKI[602]
PR AD	[1]MTELKAKGPRAPHVAGGPPSPEVGSPLLCRPAAGPFPGSQTSDTLPEVSAIPISLDGLL<br>FPRPCQGQDPSDEKTQDQQSLSDVEGAYSRAEATRGAGGSSSSPPEKDSGLLDSVLDT<br>LLAPSGPGQSQPSPPACEVTSSWCLFGPELPEDPPAAPATQRVLSPLMSRSGCKVGDSS<br>GTAAAHKVLPRGLSPARQLLLPASESPHWSGAPVKPSPQAAAVEVEEEDGSESEESAG

	PLLKGKPRALGGAAAGGGAAAVPPGAAAGGVALVPKEDSRFSAPRVALVEQDAPMAP<br>GRSPLATTVMDFIHVPILPLNHALLAARTRQLLEDESYDGGAGAASAFAPPRSSPCASS<br>TPVAVGDFPDCAYPPDAEPKDDAYPLYSDFQPPALKIKEEEEGAEASARSPRSYLVAGA<br>NPAAFPDFPLGPPPPLPPRATPSRPGEAAVTAAPASASVSSASSSGSTLECILYKAEGAPP<br>QQGPFAPPPCKAPGASGCLLPRDGLPSTSASAAAAGAAPALYPALGLNGLPQLGYQAA<br>VLKEGLPQVYPPYLNYLRPDSEASQSPQYSFESLPQKI^{566}
GR AD	^{1}MDSKESLTPGREENPSSVLAQERGDVMDFYKTLRGGATVKVSASSPSLAVASQSDSK<br>QRRLLVDFPKGSVSNAQQPDLSKAVSLSMGLYMGETETKVMGNDLGFPQQGQISLSS<br>GETDLKLLEESIANLNRSTSVPENPKSSASTAVSAAPTEKEFPKTHSDVSSEQQHLKGQ<br>TGTNGGNVKLYTTDQSTFDILQDLEFSSGSPGKETNESPWRSDLLIDENCLLSPLAGED<br>DSFLLEGNSNEDCKPLILPDTKPKIKDNGDLVLSSPSNVTLPQVKTEKEDFIELCTPGVI<br>KQEKLGTVYCQASFPGANIIGNKMSAISVHGVSTSGGQMYHYDMNTASLSQQQDQK<br>PIFNVIPPIPVGSENWNRCQGSGDDNLTSLGTLNFPGRTVFSNGYSSPSMRPDVSSPPSS<br>SSTATTGPPPKL^{420}
ERα AD	^{1}MTMTLHTKASGMALLHQIQGNELEPLNRPQLKIPLERPLGEVYLDSSKPAVYNYPEG<br>AAYEFNAAAAANAQVYGQTGLPYGPGSEAAAFGSNGLGGFPPLNSVSPSPLMLLHPP<br>PQLSPFLQPHGQQVPYYLENEPSGYTVREAGPPAFYRPNSDNRRQGGRERLASTNDK<br>GSMAMESAKETRY^{184}
Nt	^{1}MEVQLGLGRVYPRPPSKTYRGAFQNLFQSVREVIQNPGPRHPEAASAAPPGASLLLL<br>QQQQQQQQQQQQQQQQQQQQQQQETSPRQQQQQQGEDGSPQAHRRGPTGYLVLDEEQ<br>QPSQPQSALECHPERGCVPEPGAAVAASKGLPQQLPAPP^{151}
Nt C123 (C129S)	^{1}MEVQLGLGRVYPRPPSKTYRGAFQNLFQSVREVIQNPGPRHPEAASAAPPGASLLLL<br>QQQQQQQQQQQQQQQQQQQQQQQETSPRQQQQQQGEDGSPQAHRRGPTGYLVLDEEQ<br>QPSQPQSALECHPERG**S**VPEPGAAVAASKGLPQQLPAPP^{151}
Nt L26P	^{1}MEVQLGLGRVYPRPPSKTYRGAFQN**P**FQSVREVIQNPGPRHPEAASAAPPGASLLLL<br>QQQQQQQQQQQQQQQQQQQQQQQETSPRQQQQQQGEDGSPQAHRRGPTGYLVLDEEQ<br>QPSQPQSALECHPERGCVPEPGAAVAASKGLPQQLPAPP^{151}
Tau-5*	^{330}AAGSSGTLELPSTLSLYKSGALDEAAAYQSRDYYNFPLALAGPPPPPPPPHPHARIKL<br>ENPLDYGSAWAAAAAQCRYGDLASLHGAGAAGPGSGSPSAAASSSWHTLFTAEEGQ<br>LYGPC^{448}
Tau-5* C404 (C448 deleted)	^{330}AAGSSGTLELPSTLSLYKSGALDEAAAYQSRDYYNFPLALAGPPPPPPPPHPHARIKL<br>ENPLDYGSAWAAAAAQCRYGDLASLHGAGAAGPGSGSPSAAASSSWHTLFTAEEGQ<br>LYGP*^{448}
Tau-5* C404S	^{330}AAGSSGTLELPSTLSLYKSGALDEAAAYQSRDYYNFPLALAGPPPPPPPPHPHARIKL<br>ENPLDYGSAWAAAAAQ**S**RYGDLASLHGAGAAGPGSGSPSAAASSSWHTLFTAEEGQL<br>YGPC^{448}
noAro (all Tau-5* aromatic residues are substituted with A)	^{330}AAGSSGTLELPSTLSL**A**KSGALDEAAA**A**QSRD**AA**N**A**PLALAGPPPPPPPP**A**P**A**ARIK<br>LENPLD**A**GSA**A**AAAAAQCR**A**GDLASL**A**GAGAAGPGSGSPAAASSS**AA**TL**A**TAEEG<br>QL**A**GPC^{448}
h2SA (Y393S+W397A+Y406S+H413A)	^{330}AAGSSGTLELPSTLSLYKSGALDEAAAYQSRDYYNFPLALAGPPPPPPPPHPHARIKL<br>ENPLD**S**GSA**A**AAAAAQCR**S**GDLASL**A**GAGAAGPGSGSPAAASSSWHTLFTAEEGQL<br>YGPC^{448}

h3A (W433A+H434 A+F437A)	^{330}AAGSSGTLELPSTLSLYKSGALDEAAAYQSRDYYNFPLALAGPPPPPPPPHPHARIKL<br>ENPLDYGSAWAAAAAQCRYGDLASLHGAGAAGPGSGSPSAAASSS**AA**TL**A**TAEEGQ<br>LYGPC^{448}
C404Y	^{330}AAGSSGTLELPSTLSLYKSGALDEAAAYQSRDYYNFPLALAGPPPPPPPPHPHARIKL<br>ENPLDYGSAWAAAAAQ**Y**RYGDLASLHGAGAAGPGSGSPSAAASSSWHTLFTAEEGQ<br>LYGPC^{448}
AAA (G394A+S395A +G407A)	^{330}AAGSSGTLELPSTLSLYKSGALDEAAAYQSRDYYNFPLALAGPPPPPPPPHPHARIKL<br>ENPLDY**AA**AWAAAAAQCRYADL**A**SLHGAGAAGPGSGSPSAAASSSWHTLFTAEEGQ<br>LYGPC^{448}
A398P	^{330}AAGSSGTLELPSTLSLYKSGALDEAAAYQSRDYYNFPLALAGPPPPPPPPHPHARIKL<br>ENPLDYGSAW**P**AAAAQCRYGDLASLHGAGAAGPGSGSPSAAASSSWHTLFTAEEGQ<br>LYGPC^{448}
L436P	^{330}AAGSSGTLELPSTLSLYKSGALDEAAAYQSRDYYNFPLALAGPPPPPPPPHPHARIKL<br>ENPLDYGSAWAAAAAQCRYGDLASLHGAGAAGPGSGSPSAAASSSWHT**P**FTAEEGQ<br>LYGPC^{448}
T435P	^{330}AAGSSGTLELPSTLSLYKSGALDEAAAYQSRDYYNFPLALAGPPPPPPPPHPHARIKL<br>ENPLDYGSAWAAAAAQCRYGDLASLHGAGAAGPGSGSPSAAASSSWH**P**LFTAEEGQ<br>LYGPC^{448}
A398P+L436P	^{330}AAGSSGTLELPSTLSLYKSGALDEAAAYQSRDYYNFPLALAGPPPPPPPPHPHARIKL<br>ENPLDYGSAW**P**AAAAQCRYGDLASLHGAGAAGPGSGSPSAAASSSWHT**P**FTAEEGQ<br>LYGPC^{448}
A398P+T435P	^{330}AAGSSGTLELPSTLSLYKSGALDEAAAYQSRDYYNFPLALAGPPPPPPPPHPHARIKL<br>ENPLDYGSAW**P**AAAAQCRYGDLASLHGAGAAGPGSGSPSAAASSSWH**P**LFTAEEGQ<br>LYGPC^{448}
G407A	^{330}AAGSSGTLELPSTLSLYKSGALDEAAAYQSRDYYNFPLALAGPPPPPPPPHPHARIKL<br>ENPLDYGSAWAAAAAQCRY**A**DLASLHGAGAAGPGSGSPSAAASSSWHTLFTAEEGQ<br>LYGPC^{448}
Ct	^{441}EGQLYGPCGGGGGGGGGGGGGGGGGGGGGGGGEAGAVAPYGYTRPPQGLAGQES<br>DFTAPDVWYPGGMVSRVPYPSPTCVKSEMGPWMDSYSGPYGDMRLETARDHVLPID<br>YYFPPQKT^{558}
Ct 4G	^{441}EGQLYGPCGG********************GGEAGAVAPYGYTRPPQGLAGQESDFTAPD<br>VWYPGGMVSRVPYPSPTCVKSEMGPWMDSYSGPYGDMRLETARDHVLPIDYYFPPQ<br>KT^{558}
Ct C518 (C448 is deleted)	^{441}EGQLYGP*GG********************GGEAGAVAPYGYTRPPQGLAGQESDFTAPDV<br>WYPGGMVSRVPYPSPTCVKSEMGPWMDSYSGPYGDMRLETARDHVLPIDYYFPPQK<br>T^{558}
allTau-5 24G	^{330}AAGSSGTLELPSTLSLYKSGALDEAAAYQSRDYYNFPLALAGPPPPPPPPHPHARIKL<br>ENPLDYGSAWAAAAAQCRYGDLASLHGAGAAGPGSGSPSAAASSSWHTLFTAEEGQ<br>LYGPCGGGGGGGGGGGGGGGGGGGGGGGGEAGAVAPYGYTRPPQGLAGQESDFTA<br>PDVWYPGGMVSRVPYPSPTCVKSEMGPWMDSYSGPYGDMRLETARDHVLPIDYYFP<br>PQKT^{558}

allTau-5 CtoS	330AAGSSGTLELPSTLSLYKSGALDEAAAYQSRDYYNFPLALAGPPPPPPPPHPHARIKL ENPLDYGSAWAAAAAQ**S**RYGDLASLHGAGAAGPGSGPSAAASSSWHTLFTAEEGQL YGP**S**GG********************GGEAGAVAPYGYTRPPQGLAGQESDFTAPDVWYPG GMVSRVPYPSPT**S**VKSEMGPWMDSYSGPYGDMRLETARDHVLPIDYYFPPQKT558
allTau-5 CtoS PP	330AAGSSGTLELPSTLSLYKSGALDEAAAYQSRDYYNFPLALAGPPPPPPPPHPHARIKL ENPLDYGSAW**P**AAAAQ**S**RYGDLASLHGAGAAGPGSGPSAAASSSWHT**P**FTAEEGQL YGP**S**GG********************GGEAGAVAPYGYTRPPQGLAGQESDFTAPDVWYPG GMVSRVPYPSPT**S**VKSEMGPWMDSYSGPYGDMRLETARDHVLPIDYYFPPQKT558
allTau-5 C404	330AAGSSGTLELPSTLSLYKSGALDEAAAYQSRDYYNFPLALAGPPPPPPPPHPHARIKL ENPLDYGSAWAAAAAQCRYGDLASLHGAGAAGPGSGPSAAASSSWHTLFTAEEGQ LYGP**S**GG********************GGEAGAVAPYGYTRPPQGLAGQESDFTAPDVWYPG GMVSRVPYPSPT**S**VKSEMGPWMDSYSGPYGDMRLETARDHVLPIDYYFPPQKT558
allTau-5 C448	330AAGSSGTLELPSTLSLYKSGALDEAAAYQSRDYYNFPLALAGPPPPPPPPHPHARIKL ENPLDYGSAWAAAAAQ**S**RYGDLASLHGAGAAGPGSGPSAAASSSWHTLFTAEEGQL YGPCGG********************GGEAGAVAPYGYTRPPQGLAGQESDFTAPDVWYPG GMVSRVPYPSPT**S**VKSEMGPWMDSYSGPYGDMRLETARDHVLPIDYYFPPQKT558
allTau-5 C518	330AAGSSGTLELPSTLSLYKSGALDEAAAYQSRDYYNFPLALAGPPPPPPPPHPHARIKL ENPLDYGSAWAAAAAQ**S**RYGDLASLHGAGAAGPGSGPSAAASSSWHTLFTAEEGQL YGP**S**GG********************GGEAGAVAPYGYTRPPQGLAGQESDFTAPDVWYPG GMVSRVPYPSPTCVKSEMGPWMDSYSGPYGDMRLETARDHVLPIDYYFPPQKT558

***** amino acid deletion.

Table S2.

Primers used for the amplification of constructs in the LacI tethering assay.

CACCGGGTTCTGCGGGTTCTGCCGCAGGTggatccGCGATaATGACCATGACCCTCCACACCAAAGCATCTGGGATGGCC	pJM118-CFP-LacI-ER_AD_Human-FW
gcaagcttgtcgacggcgctcgaattcGGGCCCTCTAGACtcaGTAGCGAGTCTCCTTGGCAGAT	pJM118-CFP-LacI-ER_AD_Human-RV
CACCGGGTTCTGCGGGTTCTGCCGCAGGTggatccGCGATaATGGAGACCAAAGGCTACCACAGT	pJM118-CFP-LacI-NR3C2(MR)_AD_Human-FW
gcaagcttgtcgacggcgctcgaattcGGGCCCTCTAGACTCATATTTTTGAAGGTCTTGAAGATCCAGTAGAAACACTT	pJM118-CFP-LacI-NR3C2(MR)_AD_Human-RV
CACCGGGTTCTGCGGGTTCTGCCGCAGGTggatccGCGATaATGGACTCCAAAGAATCATTAACTCCTGGTAGAGAAGAAAACCCCAG	pJM118-CFP-LacI-NR3C1(GR)_AD_Human-FW
gcaagcttgtcgacggcgctcgaattcGGGCCCTCTAGACtcaGAGTTTGGGAGGTGGTCCTGT	pJM118-CFP-LacI-NR3C1(GR)_AD_Human-RV
CACCGGGTTCTGCGGGTTCTGCCGCAGGTggatccGCGATaATGACTGAGCTGAAGGCAAAGG	pJM118-CFP-LacI-PGR(PR)_AD_Human-FW
gcaagcttgtcgacggcgctcgaattcGGGCCCTCTAGACtcaAATCTTCTGAGGTAATGACTCGAAGCTG	pJM118-CFP-LacI-PGR(PR)_AD_Human-RV
CACCGGGTTCTGCGGGTTCTGCCGCAGGTggatccGCGATaATGGAAGTGCAGTTAGGGCT	pJM118-CFP-LacI-AR_AD_Human-FW
gcaagcttgtcgacggcgctcgaattcGGGCCCTCTAGACtcaGGTCTTCTGGGGTGGAAAGTAATAGTCA	pJM118-CFP-LacI-AR_AD_Human-RV
CACCGGGTTCTGCGGGTTCTGCCGCAGGTggatccGCGATaGCGTCCAATGACTACACTCAGCAG	pJM118_hFUS-LCD-FW
gcaagcttgtcgacggcgctcgaattcGGGCCCTCTAGACTCAGATGGTGTT	pJM118_hFUS-LCD-RV
CACCGGGTTCTGCGGGTTCTGCCGCAGGTggatccGCGATaATGACTGAGCTGAAGGCAAAGG	pJM118-CFP-LacI-PGR(PR)_AD_Human-WF
gcaagcttgtcgacggcgctcgaattcGGGCCCTCTAGACtcaAATCTTCTGAGGTAATGACTCGAAGCTG	pJM118-CFP-LacI-PGR(PR)_AD_Human-RV

REFERENCES

1. A. S. Holehouse, B. B. Kragelund, The molecular basis for cellular function of intrinsically disordered protein regions. *Nat. Rev. Mol. Cell Biol.* **25**, 187–211 (2024).

2. M. Biesaga, M. Frigolé-Vivas, X. Salvatella, Intrinsically disordered proteins and biomolecular condensates as drug targets. *Curr. Opin. Chem. Biol.* **62**, 90–100 (2021).

3. S. J. Metallo, Intrinsically disordered proteins are potential drug targets. *Curr. Opin. Chem. Biol.* **14**, 481–488 (2010).

4. L. Tatenhorst, K. Eckermann, V. Dambeck, L. Fonseca-Ornelas, H. Walle, T. Lopes da Fonseca, J. C. Koch, S. Becker, L. Tönges, M. Bähr, T. F. Outeiro, M. Zweckstetter, P. Lingor, Fasudil attenuates aggregation of α-synuclein in models of Parkinson's disease. *Acta Neuropathol. Commun.* **4**, 39 (2016).

5. L. Boike, A. G. Cioffi, F. C. Majewski, J. Co, N. J. Henning, M. D. Jones, G. Liu, J. M. McKenna, J. A. Tallarico, M. Schirle, D. K. Nomura, Discovery of a functional covalent ligand targeting an intrinsically disordered cysteine within MYC. *Cell Chem. Biol.* **28**, 4–13.e17 (2021).

6. D. Ban, L. I. Iconaru, A. Ramanathan, J. Zuo, R. W. Kriwacki, A small molecule causes a population shift in the conformational landscape of an intrinsically disordered protein. *J. Am. Chem. Soc.* **139**, 13692–13700 (2017).

7. G. T. Heller, F. A. Aprile, T. C. T. Michaels, R. Limbocker, M. Perni, F. S. Ruggeri, B. Mannini, T. Löhr, M. Bonomi, C. Camilloni, A. de Simone, I. C. Felli, R. Pierattelli, T. P. J. Knowles, C. M. Dobson, M. Vendruscolo, Small-molecule sequestration of amyloid-β as a drug discovery strategy for Alzheimer's disease. *Sci. Adv.* **6**, eabb5924 (2020).

8. P. Robustelli, A. Ibanez-de-Opakua, C. Campbell-Bezat, F. Giordanetto, S. Becker, M. Zweckstetter, A. C. Pan, D. E. Shaw, Molecular basis of small-molecule binding to α-synuclein. *J. Am. Chem. Soc.* **144**, 2501–2510 (2022).

9. E. De Mol, R. B. Fenwick, C. T. W. Phang, V. Buzón, E. Szulc, A. De La Fuente, A. Escobedo, J. García, C. W. Bertoncini, E. Estébanez-Perpiñá, I. J. McEwan, A. Riera, X. Salvatella, EPI-001, A compound active against castration-resistant prostate cancer, targets transactivation unit 5 of the androgen receptor. *ACS Chem. Biol.* **11**, 2499–2505 (2016).

10. N. Krishnan, D. Koveal, D. H. Miller, B. Xue, S. D. Akshinthala, J. Kragelj, M. R. Jensen, C.-M. Gauss, R. Page, M. Blackledge, S. K. Muthuswamy, W. Peti, N. K. Tonks, Targeting the disordered C terminus of PTP1B with an allosteric inhibitor. *Nat. Chem. Biol.* **10**, 558–566 (2014).

11. J.-K. Myung, C. A. Bañuelos, J. G. Fernandez, N. R. Mawji, J. Wang, A. H. Tien, Y. C. Yang, I. Tavakoli, S. Haile, K. Watt, I. J. McEwan, S. Plymate, R. J. Andersen, M. D. Sadar, An androgen receptor N-terminal domain antagonist for treating prostate cancer. *J. Clin. Invest.* **123**, 2948–2960 (2013).

12. R. J. Andersen, N. R. Mawji, J. Wang, G. Wang, S. Haile, J.-K. Myung, K. Watt, T. Tam, Y. C. Yang, C. A. Bañuelos, D. E. Williams, I. J. McEwan, Y. Wang, M. D. Sadar, Regression of castrate-recurrent prostate cancer by a small-molecule inhibitor of the amino-terminus domain of the androgen receptor. *Cancer Cell* **17**, 535–546 (2010).

13. V. Laudet, C. Hänni, J. Coll, F. Catzeflis, D. Stéhelin, Evolution of the nuclear receptor gene superfamily. *EMBO J.* **11**, 1003–1013 (1992).

14. E. R. Weikum, X. Liu, E. A. Ortlund, The nuclear receptor superfamily: A structural perspective. *Protein Sci.* **27**, 1876–1892 (2018).

15. S. Basu, P. Martínez-Cristóbal, M. Frigolé-Vivas, M. Pesarrodona, M. Lewis, E. Szulc, C. A. Bañuelos, C. Sánchez-Zarzalejo, S. Bielskutė, J. Zhu, K. Pombo-García, C. Garcia-Cabau, L. Zodi, H. Dockx, J. Smak, H. Kaur, C. Batlle, B. Mateos, M. Biesaga, A. Escobedo, L. Bardia, X. Verdaguer, A. Ruffoni, N. R. Mawji, J. Wang, J. K. Obst, T. Tam, I. Brun-Heath, S. Ventura, D. Meierhofer, J. García, P. Robustelli, T. H. Stracker, M. D. Sadar, A. Riera, D. Hnisz, X. Salvatella, Rational optimization of a transcription factor activation domain inhibitor. *Nat. Struct. Mol. Biol.* **30**, 1958–1969 (2023).

16. T. Chen, Nuclear receptor drug discovery. *Curr. Opin. Chem. Biol.* **12**, 418–426 (2008).

17. V. K. Dhiman, M. J. Bolt, K. P. White, Nuclear receptors in cancer—Uncovering new and evolving roles through genomic analysis. *Nat. Rev. Genet.* **19**, 160–174 (2018).

18. A. Boija, I. A. Klein, B. R. Sabari, A. Dall'Agnese, E. L. Coffey, A. V. Zamudio, C. H. Li, K. Shrinivas, J. C. Manteiga, N. M. Hannett, B. J. Abraham, L. K. Afeyan, Y. E. Guo, J. K. Rimel, C. B. Fant, J. Schuijers, T. I. Lee, D. J. Taatjes, R. A. Young, Transcription factors activate genes through the phase-separation capacity of their activation domains. *Cell* **175**, 1842–1855.e16 (2018).

19. A. Patil, A. R. Strom, J. A. Paulo, C. K. Collings, K. M. Ruff, M. K. Shinn, A. Sankar, K. S. Cervantes, T. Wauer, J. D. St Laurent, G. Xu, L. A. Becker, S. P. Gygi, R. V. Pappu, C. P. Brangwynne, C. Kadoch, A disordered region controls cBAF activity via condensation and partner recruitment. *Cell* **186**, 4936–4955.e26 (2023).

20. T. Zarin, B. Strome, A. N. Nguyen Ba, S. Alberti, J. D. Forman-Kay, A. M. Moses, Proteome-wide signatures of function in highly diverged intrinsically disordered regions. *eLife* **8**, e46883 (2019).

21. A. S. Holehouse, R. K. Das, J. N. Ahad, M. O. G. Richardson, R. V. Pappu, CIDER: Resources to analyze sequence-ensemble relationships of intrinsically disordered proteins. *Biophys. J.* **112**, 16–21 (2017).

22. A. Sponga, J. L. Arolas, T. C. Schwarz, C. M. Jeffries, A. Rodriguez Chamorro, J. Kostan, A. Ghisleni, F. Drepper, A. Polyansky, E. De Almeida Ribeiro, M. Pedron, A. Zawadzka-Kazimierczuk, G. Mlynek, T. Peterbauer, P. Doto, C. Schreiner, E. Hollerl, B. Mateos, L. Geist, G. Faulkner, W. Kozminski, D. I. Svergun, B. Warscheid, B. Zagrovic, M. Gautel, R. Konrat, K. Djinović-Carugo, Order from disorder in the sarcomere: FATZ forms a fuzzy but tight complex and phase-separated condensates with *α-actinin Sci. Adv.* **7**, eabg7653 (2021).

23. L. E. Wong, T. H. Kim, D. R. Muhandiram, J. D. Forman-Kay, L. E. Kay, NMR experiments for studies of dilute and condensed protein phases: Application to the phase-separating protein CAPRIN1. *J. Am. Chem. Soc.* **142**, 2471–2489 (2020).

24. N. Sanfeliu-Cerdán, F. Català-Castro, B. Mateos, C. Garcia-Cabau, M. Ribera, I. Ruider, M. Porta-de-la-Riva, A. Canals-Calderón, S. Wieser, X. Salvatella, M. Krieg, A MEC-2/stomatin condensate liquid-to-solid phase transition controls neuronal mechanotransduction during touch sensing. *Nat. Cell Biol.* **25**, 1590–1599 (2023).

25. J. Klein-Seetharaman, M. Oikawa, S. B. Grimshaw, J. Wirmer, E. Duchardt, T. Ueda, T. Imoto, L. J. Smith, C. M. Dobson, H. Schwalbe, Long-range interactions within a nonnative protein. *Science* **295**, 1719–1722 (2002).

26. S. Bielskutė, C. Garcia-Cabau, M. Frigolé-Vivas, E. Szulc, E. De Mol, M. Pesarrodona, J. García, X. Salvatella, Low amounts of heavy water increase the phase separation propensity of a fragment of the androgen receptor activation domain. *Protein Sci.* **30**, 1427–1437 (2021).

27. B. Eftekharzadeh, A. Piai, G. Chiesa, D. Mungianu, J. García, R. Pierattelli, I. C. Felli, X. Salvatella, Sequence context influences the structure and aggregation behavior of a polyQ tract. *Biophys. J.* **110**, 2361–2366 (2016).

28. L. Callewaert, N. Van Tilborgh, F. Claessens, Interplay between two hormone-independent activation domains in the androgen receptor. *Cancer Res.* **66**, 543–553 (2006).

29. A. E. Conicella, G. L. Dignon, G. H. Zerze, H. B. Schmidt, A. M. D'Ordine, Y. C. Kim, R. Rohatgi, Y. M. Ayala, J. Mittal, N. L. Fawzi, TDP-43 α-helical structure tunes liquid-liquid phase separation and function. *Proc. Natl. Acad. Sci. U.S.A.* **117**, 5883–5894 (2020).

30. G. L. Dignon, W. Zheng, Y. C. Kim, R. B. Best, J. Mittal, Sequence determinants of protein phase behavior from a coarse-grained model. *PLOS Comput. Biol.* **14**, e1005941 (2018).

31. B. He, R. T. Gampe Jr, A. J. Kole, A. T. Hnat, T. B. Stanley, G. An, E. L. Stewart, R. I. Kalman, J. T. Minges, E. M. Wilson, Structural basis for androgen receptor interdomain and

coactivator interactions suggests a transition in nuclear receptor activation function dominance. *Mol. Cell* **16**, 425–438 (2004).

32. E. De Mol, E. Szulc, C. Di Sanza, P. Martínez-Cristóbal, C. W. Bertoncini, R. B. Fenwick, M. Frigolé-Vivas, M. Masín, I. Hunter, V. Buzón, I. Brun-Heath, J. García, G. De Fabritiis, E. Estébanez-Perpiñá, I. J. McEwan, Á. R. Nebreda, X. Salvatella, Regulation of androgen receptor activity by transient interactions of its transactivation domain with general transcription regulators. *Structure* **26**, 145–152.e3 (2018).

33. B. C. Swain, P. Sarkis, V. Ung, S. Rousseau, L. Fernandez, A. Meltonyan, V. E. Aho, D. Mercadante, C. D. Mackereth, M. Aznauryan, Disordered regions of human eIF4B orchestrate a dynamic self-association landscape. *Nat. Commun.* **15**, 8766 (2024).

34. D. W. Bak, T. J. Bechtel, J. A. Falco, E. Weerapana, Cysteine reactivity across the subcellular universe. *Curr. Opin. Chem. Biol.* **48**, 96–105 (2019).

35. G. Bulaj, T. Kortemme, D. P. Goldenberg, Ionization-reactivity relationships for cysteine thiols in polypeptides. *Biochemistry* **37**, 8965–8972 (1998).

36. J. Zhu, X. Salvatella, P. Robustelli, Small molecules targeting the disordered transactivation domain of the androgen receptor induce the formation of collapsed helical states. *Nat. Commun.* **13**, 6390 (2022).

37. J. Xie, H. He, W. Kong, Z. Li, Z. Gao, D. Xie, L. Sun, X. Fan, X. Jiang, Q. Zheng, G. Li, J. Zhu, G. Zhu, Targeting androgen receptor phase separation to overcome antiandrogen resistance. *Nat. Chem. Biol.* **18**, 1341–1350 (2022).

38. F. Zhang, M. Biswas, S. Massah, J. Lee, S. Lingadahalli, S. Wong, C. Wells, J. Foo, N. Khan, H. Morin, N. Saxena, S. H. Y. Kung, B. Sun, A. K. P. Nuñez, C. Sanchez, N. Chan, L. Ung, U. B. Altıntaş, J. M. Bui, Y. Wang, L. Fazli, H. Z. Oo, P. S. Rennie, N. A. Lack, A. Cherkasov, M. E. Gleave, J. Gsponer, N. Lallous, Dynamic phase separation of the androgen receptor and its coactivators key to regulate gene expression. *Nucleic Acids Res.* **51**, 99–116 (2023).

39. L. Chen, Z. Zhang, Q. Han, B. K. Maity, L. Rodrigues, E. Zboril, R. Adhikari, S.-H. Ko, X. Li, S. R. Yoshida, P. Xue, E. Smith, K. Xu, Q. Wang, T. H.-M. Huang, S. Chong, Z. Liu, Hormone-induced enhancer assembly requires an optimal level of hormone receptor multivalent interactions. *Mol. Cell* **83**, 3438–3456.e12 (2023).

40. A. Mullard, Biomolecular condensates pique drug discovery curiosity. *Nat. Rev. Drug Discov.* **18**, 324–326 (2019).

41. R. J. Wheeler, Therapeutics-how to treat phase separation-associated diseases. *Emerg. Top. Life Sci.* **4**, 307–318 (2020).

42. S. Ambadi Thody, H. D. Clements, H. Baniasadi, A. S. Lyon, M. S. Sigman, M. K. Rosen, Small-molecule properties define partitioning into biomolecular condensates. *Nat. Chem.* **16**, 1794–1802 (2024).

43. H. R. Kilgore, P. G. Mikhael, K. J. Overholt, A. Boija, N. M. Hannett, C. Van Dongen, T. I. Lee, Y.-T. Chang, R. Barzilay, R. A. Young, Distinct chemical environments in biomolecular condensates. *Nat. Chem. Biol.* **20**, 291–301 (2024).

44. S. M. Janicki, T. Tsukamoto, S. E. Salghetti, W. P. Tansey, R. Sachidanandam, K. V. Prasanth, T. Ried, Y. Shav-Tal, E. Bertrand, R. H. Singer, D. L. Spector, From silencing to gene expression: Real-time analysis in single cells. *Cell* **116**, 683–698 (2004).

45. S. Chong, C. Dugast-Darzacq, Z. Liu, P. Dong, G. M. Dailey, C. Cattoglio, A. Heckert, S. Banala, L. Lavis, X. Darzacq, R. Tjian, Imaging dynamic and selective low-complexity domain interactions that control gene transcription. *Science* **361**, eaar2555 (2018).

46. H. Lyons, R. T. Veettil, P. Pradhan, C. Fornero, N. De La Cruz, K. Ito, M. Eppert, R. G. Roeder, B. R. Sabari, Functional partitioning of transcriptional regulators by patterned charge blocks. *Cell* **186**, 327–345.e28 (2023).

47. S. Basu, S. D. Mackowiak, H. Niskanen, D. Knezevic, V. Asimi, S. Grosswendt, H. Geertsema, S. Ali, I. Jerković, H. Ewers, S. Mundlos, A. Meissner, D. M. Ibrahim, D. Hnisz, Unblending of transcriptional condensates in human repeat expansion disease. *Cell* **181**, 1062–1079.e30 (2020).

48. Y. E. Guo, J. C. Manteiga, J. E. Henninger, B. R. Sabari, A. Dall'Agnese, N. M. Hannett, J.-H. Spille, L. K. Afeyan, A. V. Zamudio, K. Shrinivas, B. J. Abraham, A. Boija, T.-M. Decker, J. K. Rimel, C. B. Fant, T. I. Lee, I. I. Cisse, P. A. Sharp, D. J. Taatjes, R. A. Young, Pol II phosphorylation regulates a switch between transcriptional and splicing condensates. *Nature* **572**, 543–548 (2019).

49. D. Flores-Solis, I. P. Lushpinskaia, A. A. Polyansky, A. Changiarath, M. Boehning, M. Mirkovic, J. Walshe, L. M. Pietrek, P. Cramer, L. S. Stelzl, B. Zagrovic, M. Zweckstetter, Driving forces behind phase separation of the carboxy-terminal domain of RNA polymerase II. *Nat. Commun.* **14**, 5979 (2023).

50. G. Pei, X. Wang, T. Li, P. Li, Sequence composition dictates condensate miscibility. bioRxiv 626135 [Preprint] (2024). https://doi.org/10.1101/2024.11.29.626135.

51. A. C. Murthy, W. S. Tang, N. Jovic, A. M. Janke, D. H. Seo, T. M. Perdikari, J. Mittal, N. L. Fawzi, Molecular interactions contributing to FUS SYGQ LC-RGG phase separation and co-partitioning with RNA polymerase II heptads. *Nat. Struct. Mol. Biol.* **28**, 923–935 (2021).

52. V. F. Thompson, R. A. Victor, A. A. Morera, M. Moinpour, M. N. Liu, C. C. Kisiel, K. Pickrel, C. E. Springhower, J. C. Schwartz, Transcription-dependent formation of nuclear granules containing FUS and RNA pol II. *Biochemistry* **57**, 7021–7032 (2018).

53. A. Bremer, M. Farag, W. M. Borcherds, I. Peran, E. W. Martin, R. V. Pappu, T. Mittag, Deciphering how naturally occurring sequence features impact the phase behaviours of disordered prion-like domains. *Nat. Chem.* **14**, 196–207 (2022).

54. A. Kuzmanic, G. R. Bowman, J. Juarez-Jimenez, J. Michel, F. L. Gervasio, Investigating cryptic binding sites by molecular dynamics simulations. *Acc. Chem. Res.* **53**, 654–661 (2020).

55. Y. Chebaro, A. J. Ballard, D. Chakraborty, D. J. Wales, Intrinsically disordered energy landscapes. *Sci. Rep.* **5**, 10386 (2015).

56. X. Liu, A. Ciulli, Proximity-based modalities for biology and medicine. *ACS Cent. Sci.* **9**, 1269–1284 (2023).

57. J. Kyte, R. F. Doolittle, A simple method for displaying the hydropathic character of a protein. *J. Mol. Biol.* **157**, 105–132 (1982).

58. R. K. Das, R. V. Pappu, Conformations of intrinsically disordered proteins are influenced by linear sequence distributions of oppositely charged residues. *Proc. Natl. Acad. Sci. U.S.A.* **110**, 13392–13397 (2013).

59. E. W. Martin, A. S. Holehouse, I. Peran, M. Farag, J. J. Incicco, A. Bremer, C. R. Grace, A. Soranno, R. V. Pappu, T. Mittag, Valence and patterning of aromatic residues determine the phase behavior of prion-like domains. *Science* **367**, 694–699 (2020).

60. A. S. Holehouse, G. M. Ginell, D. Griffith, E. Böke, Clustering of aromatic residues in prion-like domains can tune the formation, state, and organization of biomolecular condensates. *Biochemistry* **60**, 3566–3581 (2021).

61. M. C. Cohan, M. K. Shinn, J. M. Lalmansingh, R. V. Pappu, Uncovering non-random binary patterns within sequences of intrinsically disordered proteins. *J. Mol. Biol.* **434**, 167373 (2022).

62. *Dodo: DODO Is a Python Package for Taking an AF2 Structure and Redesigning the IDRs to Look More "IDR like"* (Github); https://github.com/idptools/dodo?tab=readme-ov-file.

63. M. Kar, A. E. Posey, F. Dar, A. A. Hyman, R. V. Pappu, Glycine-rich peptides from FUS have an intrinsic ability to self-assemble into fibers and networked fibrils. *Biochemistry* **60**, 3213–3222 (2021).

64. V. Y. Orekhov, V. A. Jaravine, Analysis of non-uniformly sampled spectra with multi-dimensional decomposition. *Prog. Nucl. Magn. Reson. Spectrosc.* **59**, 271–292 (2011).

65. F. Delaglio, S. Grzesiek, G. W. Vuister, G. Zhu, J. Pfeifer, A. Bax, NMRPipe: A multidimensional spectral processing system based on UNIX pipes. *J. Biomol. NMR* **6**, 277–293 (1995).

66. W. F. Vranken, W. Boucher, T. J. Stevens, R. H. Fogh, A. Pajon, M. Llinas, E. L. Ulrich, J. L. Markley, J. Ionides, E. D. Laue, The CCPN data model for NMR spectroscopy: Development of a software pipeline. *Proteins* **59**, 687–696 (2005).

67. Z. Solyom, M. Schwarten, L. Geist, R. Konrat, D. Willbold, B. Brutscher, BEST-TROSY experiments for time-efficient sequential resonance assignment of large disordered proteins. *J. Biomol. NMR* **55**, 311–321 (2013).

68. R. Weisemann, H. Rüterjans, W. Bermel, 3D triple-resonance NMR techniques for the sequential assignment of NH and 15N resonances in 15N- and 13C-labelled proteins. *J. Biomol. NMR* **3**, 113–120 (1993).

69. S. Mori, C. Abeygunawardana, M. O. Johnson, P. C. M. Vanzijl, Improved sensitivity of HSQC spectra of exchanging protons at short interscan delays using a new fast HSQC (FHSQC) detection scheme that avoids water saturation. *J. Magn. Reson. B* **108**, 94–98 (1995).

70. M. Piotto, V. Saudek, V. Sklenár, Gradient-tailored excitation for single-quantum NMR spectroscopy of aqueous solutions. *J. Biomol. NMR* **2**, 661–665 (1992).

71. C. Camilloni, A. De Simone, W. F. Vranken, M. Vendruscolo, Determination of secondary structure populations in disordered states of proteins using nuclear magnetic resonance chemical shifts. *Biochemistry* **51**, 2224–2231 (2012).

72. K. S. Jensen, J. T. Pedersen, J. R. Winther, K. Teilum, The pKa value and accessibility of cysteine residues are key determinants for protein substrate discrimination by glutaredoxin. *Biochemistry* **53**, 2533–2540 (2014).

73. J. A. Marsh, J. D. Forman-Kay, Sequence determinants of compaction in intrinsically disordered proteins. *Biophys. J.* **98**, 2383–2390 (2010).

74. J. Naderi, A. P. Magalhaes, G. Kibar, G. Stik, Y. Zhang, S. D. Mackowiak, H. M. Wieler, F. Rossi, R. Buschow, M. Christou-Kent, M. Alcoverro-Bertran, T. Graf, M. Vingron, D. Hnisz, An activity-specificity trade-off encoded in human transcription factors. *Nat. Cell Biol.* **26**, 1309–1321 (2024).

75. J. Schindelin, I. Arganda-Carreras, E. Frise, V. Kaynig, M. Longair, T. Pietzsch, S. Preibisch, C. Rueden, S. Saalfeld, B. Schmid, J.-Y. Tinevez, D. J. White, V. Hartenstein, K. Eliceiri, P. Tomancak, A. Cardona, Fiji: An open-source platform for biological-image analysis. *Nat. Methods* **9**, 676–682 (2012).

76. S. Alberti, S. Saha, J. B. Woodruff, T. M. Franzmann, J. Wang, A. A. Hyman, A user's guide for phase separation assays with purified proteins. *J. Mol. Biol.* **430**, 4806–4820 (2018).

77. G. Koulouras, A. Panagopoulos, M. A. Rapsomaniki, N. N. Giakoumakis, S. Taraviras, Z. Lygerou, EasyFRAP-web: A web-based tool for the analysis of fluorescence recovery after photobleaching data. *Nucleic Acids Res.* **46**, W467–W472 (2018).

78. E. W. Martin, A. S. Holehouse, C. R. Grace, A. Hughes, R. V. Pappu, T. Mittag, Sequence determinants of the conformational properties of an intrinsically disordered protein prior to and upon multisite phosphorylation. *J. Am. Chem. Soc.* **138**, 15323–15335 (2016).
